# Polymorphic Phase Transformations in Nanocrystalline Ag_2_S Silver Sulfide in a Wide Temperature Interval and Influence of Nanostructured Ag_2_S on the Interface Formation in Ag_2_S/ZnS Heteronanostructure

**DOI:** 10.3390/nano12101668

**Published:** 2022-05-13

**Authors:** Albina A. Valeeva, Stanislav I. Sadovnikov, Aleksandr I. Gusev

**Affiliations:** 1 Institute of Solid State Chemistry, Ural Branch of the Russian Academy of Sciences, 620990 Ekaterinburg, Russia; anibla_v@mail.ru (A.A.V.); sadovnikov@ihim.uran.ru (S.I.S.); 2 Ural Federal University named after the first President of Russia B. N. Yeltsin, 620002 Ekaterinburg, Russia

**Keywords:** sulfides, silver sulfide, *α*-Ag_2_S acanthite, *β*-Ag_2_S argentite, *γ*-Ag_2_S phase, crystal structures, phase transformations, zinc sulfide ZnS, Ag_2_S/ZnS heteronanostructures, interface, elastic properties anisotropy

## Abstract

Phase transformations that take place in nanocrystalline Ag_2_S silver sulfide have been systematically studied at temperatures from 298 to 893 K. The crystal structures of the polymorphic modifications *α*-Ag_2_S, *β*-Ag_2_S, and *γ*-Ag_2_S of nanocrystalline Ag_2_S have been found. It is established that the interstitial spacings between ions of silver in the superionic phases *β*-Ag_2_S and *γ*-Ag_2_S are noticeably smaller than diameter of the Ag^+^ ion. As a result of which, the probabilities of filling the sites of the metal sublattices of these phases with Ag atoms are very small. It was found that the “*α*-Ag_2_S—*β*-Ag_2_S” and “*β*-Ag_2_S—*γ*-Ag_2_S” transitions between polymorphic modifications of silver sulfide occur as phase transformations of the first order at temperatures of ~440–442 K and ~850–860 K. The structure of interface forming by nanostructured Ag_2_S and ZnS is considered, taking into account the anisotropy of elastic properties of these sulfides. It is established that a large amount of cubic zinc sulfide stabilizes the cubic structure of *β*-Ag_2_S argentite at 300 K during the co-deposition of Ag_2_S/ZnS heteronanostructures from colloid solutions. It is found that placing Ag atoms at four crystallographic positions located in one plane of the unit cell of cubic *β*-Ag_2_S argentite is most favorable for the appearance of Ag_2_S/ZnS heterostructures. The smallest strain distortions at the interface are observed at the minimum difference of shear moduli of the components forming heteronanostructure. The distributions of elastic characteristics, including the shear moduli of monocrystalline particles of cubic *β*-Ag_2_S argentite and ZnS sphalerite from the [*hkl*] direction, are found. The formation of Ag_2_S/ZnS heteronanostructures, in which the interface is formed by the (*hk*0) ≡ (110) plane of ZnS sphalerite and the (*hk* 0.4123) ≡ (1 1 0.4123) plane of *β*-Ag_2_S argentite, is the most energetically favorable.

## 1. Introduction

Among binary semiconductor compounds, such compounds of metals with sulfur as sulfides CuS, Cu_2_S, Ag_2_S, ZnS, CdS, HgS, SnS, and PbS occupy an important place. These sulfides are used in various fields of modern technology.

Semiconductor sulfide nanostructures represent the promising group of nanocrystalline materials for various fields of application [1,2,3]. Silver sulfide Ag_2_S attracts a lot of attention [4,5,6]. Silver sulfide is a semiconductor at temperature < ~420–450 K, and this sulfide possesses a superionic conductivity at temperature more than 452 K. Nanocrystalline silver sulfide is the versatile semiconductor for applications in different optoelectronic devices such as photocells, photoconductors, and infrared detectors [6,7,8].

The use of nanocrystalline silver sulfide is promising for creating Ag_2_S/Ag heteronanostructures intended for operation in memory devices and resistance switches. Their action is based on the transformation of *α*-Ag_2_S acanthite into *β*-Ag_2_S argentite and the formation of a conducting channel from silver Ag and superionic *β*-Ag_2_S argentite [9,10,11,12].

Ag_2_S and ZnS are among the most sought-after semiconductor sulfides [5,13]. Recently, much attention has been attracted by heteronanostructures based on semiconductor Ag_2_S and ZnS sulfides [14,15,16,17,18,19,20,21]. Composite heteronanostructures can exhibit enhanced nonlinear phenomena in optical properties due to the quantum size effect of nanoparticles. The light emission properties of such heteronanostructures are important for their applications in optical imaging, sensors, or lasers. Composite heteronanostructures represent an interesting class of materials because their applications are of multidisciplinary importance. The main hindrance for zinc sulfide widespread use is the low emission properties of pure ZnS nanoparticles. Formation of the heteronanostructures is one way that has been used to improve the emission properties of pure ZnS in recent years. For example, in study [17], it was reported that the light emission properties of double-shell ZnS-Ag_2_S hollow nanoparticles are maximum compared to those of pure ZnS nanoparticles, and these properties highly depend on the external ZnS layer thickness. Ag_2_S/ZnS type heteronanostructures make it possible to control the band gap and are considered promising nanomaterials for solid-state ultraviolet lasers and fast-acting resistive switches as well as for catalysis.

In Ag_2_S/ZnS heteronanostructures, an important role belongs to the interface between silver and zinc sulfides. Strain distortions at the interface should be minimal.

Information on the elastic characteristics of ZnS and Ag_2_S is needed to assess the strain (deformation) distortions at the interface between silver and zinc sulfides. The elastic properties of cubic ZnS are well known [22,23,24,25,26,27,28,29,30,31,32]. The theoretical data on the elastic properties of semiconductor monocrystalline particles of silver sulfide Ag_2_S with monoclinic (space group No. 11—*P*2_1_/*m*) and orthorhombic (space group No. 63—*Cmcm*) structures are available in the form of databases on the websites [33,34]. According to [33], the maximal value of the Young’s modulus of modeling monoclinic (space group *P*2_1_/*m*) silver sulfide is equal to ~37.9 GPa. According to data [34], the maximal Young’s modulus *E* of orthorhombic (space group *Cmcm*) Ag_2_S is ~48.1 GPa. The elastic constants are calculated by the ab initio method, described in general terms in the study [35].

In electronics, the potentially most applicable superionic is *β*-Ag_2_S argentite, which has a body-centered cubic (bcc) (space group $Im\bar{3}m$) lattice. Recently, the thermal expansion and heat capacity of coarse-crystalline and nanocrystalline silver sulfide were measured in the temperature range 300–930 K [36,37,38] including the region of existence of argentite. The elastic properties of cubic (space group $Im\bar{3}m$) superionic *β*-Ag_2_S argentite were determined for a wide temperature range in studies [39,40].

Unfortunately, intensive studies of the synthesis and properties of nanocrystalline sulfides are not accompanied, as a rule, by a detailed study of the crystal structure. Many data on the crystal structure of coarse-crystalline sulfides were obtained back in the 1950s–1970s of the last century and even earlier on samples of natural minerals. The structure of artificial (synthetic) silver sulfides, especially nanocrystalline ones, can differ markedly from that of natural minerals in terms of unit cell parameters and even in the arrangement of atoms at other crystallographic positions. For example, the X-ray diffraction (XRD) reflections not related to the structure of monoclinic acanthite were detected in the XRD pattern of Ag_2_S nanoparticles [41].

Ag_2_S is one of a few sulfides that have three polymorphous modifications: Semiconductor monoclinic *α*-Ag_2_S acanthite, superionic argentite *β*-Ag_2_S with body centered cubic (bcc) structure, and superionic face centered cubic (fcc) *γ*-Ag_2_S phase. Application of silver sulfide in microelectronic devices is based on the reversible transition “*α*-Ag_2_S *β*-Ag_2_S”. Therefore, an accurate determination and refinement of the structure of different phases is especially important in the study of phase transformations in silver sulfide, for the use of silver sulfide in devices operating in a wide temperature range.

Studies of bulk silver sulfide, carried out from the 1930s of the last century to the beginning of the XXI century, are described in detail in the monograph [6]. Blanton et al. [42] made the first modern determination of the crystal structure of all three phases for coarse-crystalline synthetic powders of silver sulfide. Prepared silver sulfide Ag_2_S powder was studied by XRD at temperatures of 293, 523, and 923 K.

A kinetic investigation of phase transitions in bulk silver sulfide Ag_2_S by the differential thermal analysis was carried out by Živković et al. [43]. According to [43], the temperatures of phase transitions *α*-Ag_2_S—*β*-Ag_2_S и *β*-Ag_2_S—*γ*-Ag_2_S are equal to 458 and 867 K, respectively. However, no study of the structure of Ag_2_S different phases was performed in work [43].

Il’inskii et al. [44] studied the phase transition from the monoclinic semiconducting to the bcc superionic state in thin films of silver sulfide by dielectric spectroscopy in the temperature region 273–473 K. According to [44], the temperature dependences of the dielectric loss tangent of the Ag_2_S film at 425 K exhibit a hysteresis corresponding to the taking place reversible “semiconductor—superionic phase” transition. In work [44], experimental data related to the structure of the studied films of silver sulfide are absent.

Recently, the authors of work [45] studied the phase transformations in silver sulfide with sheet-like morphology under high pressure up to 30 GPa. Ran Liu et al. have found two different structural phase transitions. The first transition from monoclinic (space group *P*2_1_/*n*) acanthite *α*-Ag_2_S to orthorhombic (space group *P*2_1_2_1_2_1_) phase II is observed under the pressure of 8.9 GPa. The second transformation of orthorhombic phase II to monoclinic phase III, isosymmetric to a phase I, takes place under the pressure of 12.4 GPa. Unfortunately, the structure of the Ag_2_S monoclinic phases is described in the outdated space group *P*2_1_/*n*, therefore the convergence of the experiment and calculation is low: the Rietveld factors *R*_p_ and *ωR*_p_ are equal to 14–20% and 30–31%, respectively [45].

Thus, studies of phase transformations in silver sulfide have been carried out in few works, moreover, on coarse-crystalline samples. In a number of works, the crystal structure of the phases of Ag_2_S was not studied at all or was not exactly determined.

In works [14,16,17,18,19,21] devoted to the synthesis and study of heteronanostructures based on semiconductor Ag_2_S and ZnS sulfides, the main attention is paid to the producing conditions and optoelectronic properties of synthesized Ag_2_S/ZnS heteronanostructures, whereas the structure of the interfaces in general was not being discussed. However, the properties of heteronanostructures strongly depend on the structure of the interfaces [46,47], and on the elastic properties of nanoparticles [48]. The structure of Ag_2_S/ZnS heteronanostructures was analyzed, taking into account the morphology of Ag_2_S and ZnS sulfide monocrystalline particles in work [49] using the outdated Hartman–Perdok theory [50]. Additionally, the structure of Ag_2_S/ZnS heteronanostructures was considered in work [51] without analysis of possible mutual positions of planes of *β*-Ag_2_S argentite and ZnS sphalerite on their interface.

In this regard, in present study, an in situ systematic investigation of polymorphic phase transitions in nanocrystalline silver sulfide is performed in a wide temperature interval from 298 to 893 K, where semiconductor monoclinic *α*-Ag_2_S acanthite, superionic bcc *β*-Ag_2_S argentite, and superionic fcc *γ*-Ag_2_S phase exist. X-ray diffraction over a wide temperature range and high-resolution transmission electron microscopy (HRTEM) are used as the main experimental methods.

The other aims of this work are the following: (1) to determine possible combinations of crystallographic planes of cubic *β*-Ag_2_S argentite and ZnS sphalerite, which can physically form the interface of the Ag_2_S/ZnS heteronanostructure, and (2) to study the structure of the interface between zinc and silver sulfides, taking into account the structural features and elastic characteristics of the monocrystalline cubic *β*-Ag_2_S argentite and ZnS sphalerite.

## 2. Materials and Methods

### 2.1. Samples

Silver sulfide Ag_2_S, in a form of colloid solution and powder, was prepared by hydrochemical bath deposition, i.e., the chemical deposition from aqueous solutions. It is a conversant universal technique for preparing colloid solutions, coarse-crystalline, and nanocrystalline powders of silver sulfide [52].

Colloid solutions and powders of Ag_2_S were produced by deposition from aqueous solutions of AgNO_3_ (ACS reagent, ≥99.0%, Sigma-Aldrich, St. Louis, MO, USA), Na_2_S (ACS reagent, ≥98.0%, Sigma-Aldrich, St. Louis, MO, USA), and sodium citrate Na_3_C_6_H_5_O_7_ (Na_3_Cit) (ACS reagent, Merck KGaA, Darmstadt, Germany) using the same chemical reactants by altering their concentration in a solution. The synthesis of silver sulfide was carried out at pH ≈ 7 in the dark by the following reaction scheme:

(1) $$2{\mathrm{AgNO}}_{3}+(1+\delta ){\mathrm{Na}}_{2}S\;\overset{{\mathrm{Na}}_{3}C_{6}H_{5}O_{7}}{\to }\;{\mathrm{Ag}}_{2}S+2{\mathrm{NaNO}}_{3}$$

with an excess 0.01 ≥ *δ* ≥ 0.5 of sodium sulfide Na_2_S. An excess of Na_2_S ensures producing silver sulfide without Ag impurity.

The concentrations of main reactants AgNO_3_, Na_2_S, and Na_3_Cit were 0.625, 0.313, and 1.25 mmol·L^−1^ for the preparation of a stable colloid Ag_2_S solution, and 50, 50, and 100 mmol·L^−1^ for deposition of Ag_2_S powder. The average size of colloid nanoparticles of silver sulfide was ~4 ± 2 nm, the average particle size for powder was ~90 ± 10 nm. According to XRD data, the synthesized colloid nanoparticles and silver sulfide powder at room temperature had a monoclinic (space group *P*2_1_/*c*) crystal structure of *α*-Ag_2_S acanthite.

In our work, Ag_2_S powders with particles smaller than 50 nm are believed to be nanocrystalline, and powders with particle of about 100 and 200 nm are called fine-dispersed and coarse-crystalline powders, respectively. The procedure for the synthesis of Ag_2_S powders and colloid solutions is described in detail in [52,53,54,55].

Heteronanostructures Ag_2_S/ZnS have been produced by two-stage synthesis in detail, described in work [19]. First, silver sulfide Ag_2_S was synthesized by chemical deposition from aqueous solutions of AgNO_3_ and Na_2_S in the presence of sodium citrate Na_3_Cit. Next, Trilon B (ACS reagent, 99.0–101.0%, Sigma-Aldrich, St. Louis, MO, USA) was added to an aqueous Zn(NO_3_)_2_ (reagent grade, 98%, Sigma-Aldrich, St. Louis, MO, USA) solution with a sodium sulfide solution. The use of Trilon B promoted the appearance of nanostructured zinc sulfide on a surface of silver sulfide nanoparticles. Thus, Ag_2_S/ZnS heteronanostructures of core-shell type were formed.

### 2.2. Experimental Techniques

Primary certification of synthesized Ag_2_S colloid solution and deposited silver and zinc sulfides powders was carried out by X-ray diffraction (XRD) at 298 K in Cu*Kα*_1_-radiation in the interval of angles 2*θ* from 20 to 95° with a step 0.02°. The determining parameters of lattice and refinement of structure of the prepared colloid nanoparticles and silver sulfide powder were made using the X’Pert HighScore Plus program (version 2.2e (2.2.5); © 2009 PANalytical, B.V.: Almedo, The Netherlands) [56].

Phase transformations in silver sulfide in a temperature region 298–893 K were studied by in situ high-temperature XRD in filtered Cu*Kα* radiation using a high-resolution “Empyrean” diffractometer (X’Pert PRO MRD, PANAlytical, The Netherlands) supplied with an Anton Paar HTK-1200 Oven furnace (Graz, Austria). The diffraction measurements were made in high vacuum 0.01 Pa (7.7 × 10^−5^ mm Hg) in the interval of angles 2*θ* from 20 to 60° with a step 0.026°. The cylindrical sample in 10.0 mm diameter and 1.5 mm thickness, produced by uniaxial pressing of the synthesized silver sulfide powder in a steel compression mold, was used for measurements. Pressure of pressing was equal to 20 MPa. The surface of the sample was polished before XRD measurements.

A scanning JEOL-JSM LA 6390 electron microscope (JEOL Ltd., Tokyo, Japan) equipped with a JED 2300 Energy Dispersive X-ray Analyzer (JEOL Ltd., Tokyo, Japan) has been used for direct observation of the changes at heating and cooling of silver sulfide pressed sample.

The microstructure, particle size, and element chemical composition of sulfide powders were studied by the high-resolution transmission electron microscopy (HRTEM) on a JEOL JEM-2010 microscope (JEOL Ltd., Tokyo, Japan) with 0.14 nm lattice resolution. The elemental chemical composition of heteronanoparticles was studied on the same microscope with the use of an Phoenix (EDAX) Energy Dispersive Spectrometer (AMETEK Inc. Berwyn, PA, USA), with a Si(Li) detector having an energy resolution of 130 eV.

The method of HRTEM has been used for investigation of transformations in colloid silver sulfide nanoparticles. The HRTEM images were registered by a Tecnai G2 30 Twin microscope (FEI company, Hillsboro, OR, USA) with 0.14 nm lattice resolution. For study, colloid solution of silver sulfide was placed on the perforated carbon substrates which are fixed on copper grids. After deposition, the substrates with nanoparticles were dried and placed in a microscope on a heated holder.

In situ XRD and HRTEM measurements were performed in the “Test Centre of Nanotechnologies and Perspective Materials” of the Institute of Metal Physics (Ural Branch of Russian Academy of Sciences).

## 3. Results and Discussion

The XRD patterns of prepared fine-dispersed powder of Ag_2_S silver sulfide were recorded at temperatures of 298, 353, 423, 443, 453, 463, 523, 573, 623, 643, 723, 773, 843, 863, and 893 K. A preliminary analysis has shown that silver sulfide is monoclinic *α*-Ag_2_S at a temperature of 298–423 K, it has bcc structure of *β*-Ag_2_S argentite at a temperature from 443 to 843 K, and at a temperature of 863–893 K it has fcc crystal structure of *γ*-Ag_2_S phase.

### 3.1. α-Ag_2_S Acanthite

**Figure 1:**
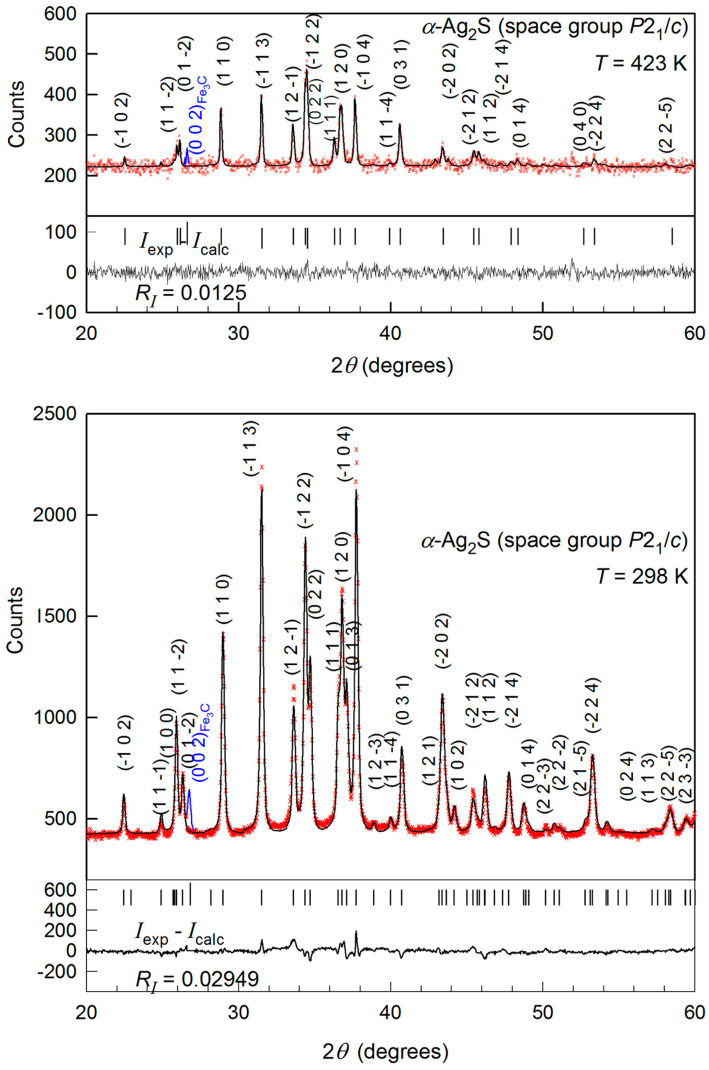
Experimental (**×**), calculated (—), and difference (*I*_exp_—*I*_calc_) XRD patterns of Ag_2_S pressed pill at temperatures of 298 and 423 K. According to refinement, Ag_2_S at 298 and 423 K is monoclinic (space group P2_1_/c) α-Ag_2_S acanthite.

The XRD patterns of pressed sample at 298 and 423 K are presented in Figure 1. Quantitative refinement of structure of synthesized powder has shown that silver sulfide has monoclinic (space group *P*2_1_/*c*) symmetry of acanthite *α*-Ag_2_S in the temperature region 298–423 K.

Experimental, calculated, and difference (*I*_exp_—*I*_calc_) XRD patterns of synthesized fine-dispersed silver sulfide powder are presented in Figure 1. The refinement of structure at 298 K made it possible to achieve a high convergence of experiment and calculation: the Rietveld factors *R*_p_, *ωR*_p_, *R_I_* (*R*_B_), and *R*_expect_ are equal to 3.218%, 4.147%, 2.949%, and 4.374%, respectively. The parameters of the monoclinic unit cell of fine-dispersed Ag_2_S powder and the atomic coordinates in this unit cell at 298 K are given in Table 1. These magnitudes are close to those found in work [53,57] for bulk Ag_2_S.

Some difference in the experimental and calculated intensities of diffraction reflections (Figure 1) is caused by the presence of texture in pressed sample caused by uniaxial pressure during powder pressing. In XRD patterns, in addition to the main monoclinic phase, there is only the reflection of the impurity phase at 2*θ* ~26.75°, corresponding to the (002) reflection of orthorhombic cementite Fe_3_C [58,59]. The appearance of the impurity is associated with the pressing of Ag_2_S powder in a steel compression mold. The amount of the impurity phase is noticeably less than 1 wt. %.

The stability of *α*-Ag_2_S acanthite is due to the mutual arrangement of Ag atoms at distances exceeding the covalent diameter of silver atom, equal to ~0.2883 nm [60]. At a temperature of 298 K, the interatomic spacing Ag1-Ag1 in monoclinic acanthite *α*-Ag_2_S is 0.33464 nm, the spacing Ag1-Ag2 changes from 0.30758 to 0.31850 nm, and the interatomic distance Ag2-Ag2 is 0.31144 nm.

Minimal interatomic spacings S-Ag1 in monoclinic *α*-Ag_2_S at 423 K are in the range from 0.24801 to 0.25231 nm, and spacings S-Ag2 change from 0.25201 to 0.27126 nm. The interatomic spacing Ag1-Ag1 in *α*-Ag_2_S at 423 K is equal to 0.33781 nm, and interatomic spacings Ag1-Ag2 change from 0.30959 to 0.32010 nm. Thus, atoms of Ag in monoclinic acanthite at a temperature of 298–423 K are at rather large distances from each other (greater than the atomic diameter of silver). Therefore, Ag and S atoms fill their crystallographic positions in monoclinic acanthite with a probability equal to 1.

Temperature increasing from 298 to 423 K leads to a slight growth of unit cell volume of monoclinic acanthite *α*-Ag_2_S from ~0.2274 to ~0.2288 nm^3^.

### 3.2. β-Ag_2_S Argentite

**Figure 2:**
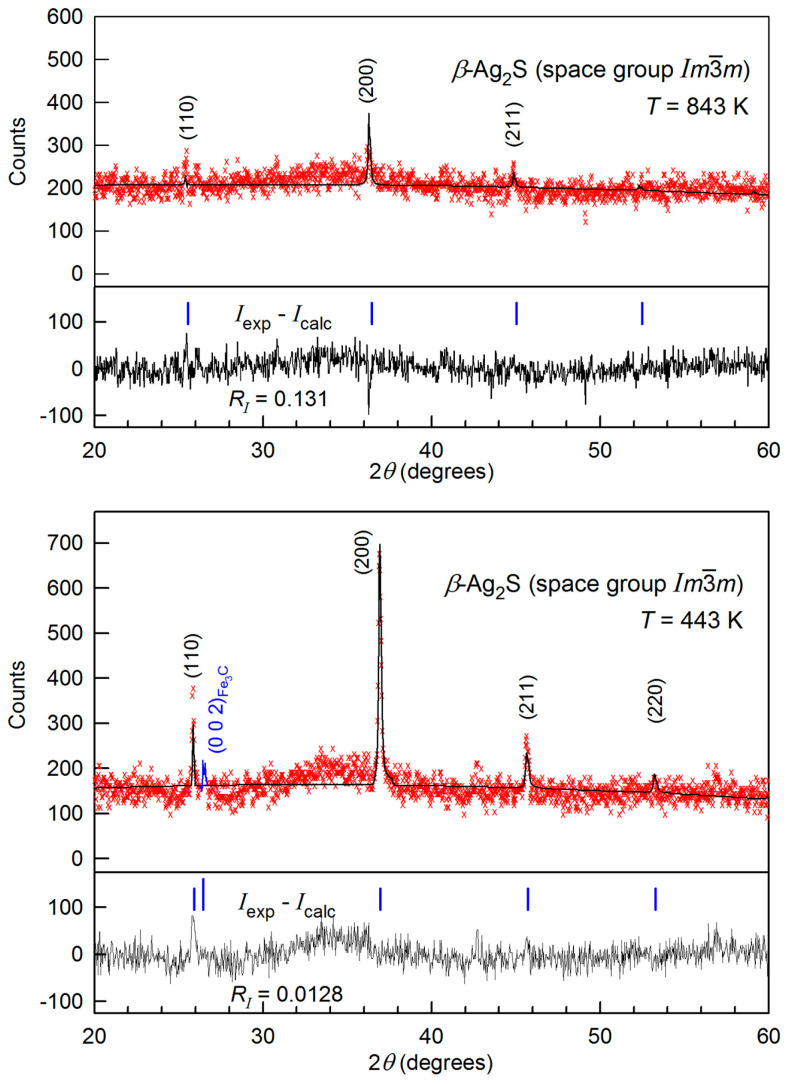
Experimental (**×**), calculated (—), and difference (*I*_exp_—*I*_calc_) XRD patterns of Ag_2_S pressed pill at temperatures of 443 and 843 K (According to the structure refinement, studied silver sulfide has bcc (space group $Im\bar{3}m$) structure of *β*-Ag_2_S argentite).

Diffraction reflections of the body-centered cubic (space group $Im\bar{3}m$) *β*-Ag_2_S phase with an argentite structure are observed in XRD patterns at higher temperatures of 443–843 K (Figure 2). Thus, the monoclinic *α*-Ag_2_S acanthite transforms into argentite at a temperature of ~440–442 K. According to the quantitative full-profile refinement of XRD pattern of silver sulfide registered at 443 K (Figure 2), the experiment and calculation converge satisfactory: Rietveld factors are *R*_p_ = 22.34%, *ωR*_p_ = 29.42%, *R_I_* (*R*_B_) = 1.28%, and *R*_expect_ = 15.69%. The parameters of bcc (space group $Im\bar{3}m$) unit cell of fine-dispersed powder of silver sulfide and the atomic coordinates in this unit cell at 443 K are given in Table 2.

The unit cell of *β*-Ag_2_S involves two formula units Ag_2_S. S atoms fill the sites of a bcc sublattice with a probability equal to 1. Four Ag atoms are randomly placed in 54 sites, 6(*b*) and 48(*j*), with small filling probabilities ~0.0974 and ~0.0716 (Table 2). Within measurement errors, these filling probabilities coincide with the occupation probabilities 0.0978 and 0.0711 of 6(*b*) and 48(*j*) argentite sites by Ag atoms, which were found in study [61]. As a result, Ag atoms are in permanent motion over these 54 possible crystallographic sites of metallic sublattice of argentite, providing superionic conductivity of argentite. According to [11,62,63], the conductivity of supercooled argentite *β*-Ag_2_S at room temperature is (1.3–1.6) × 10^3^ Ohm^−1^·cm^−1^, which is about 6 × 10^5^ times larger than the conductivity 2.5 × 10^−3^ Ohm^−1^·cm^−1^ of monoclinic phase.

In cubic superionic phases, ions Ag^+^ are surrounded by six sites of nonmetal sublattice. The ionic diameter of Ag^+^ ion for coordination number 6 is ~0.252 nm [60]. The interstitial distances between silver ions in bcc *β*-Ag_2_S argentite at 443 K are significantly less than the interatomic distances in monoclinic *α*-Ag_2_S acanthite at a close temperature of 423 K. In *β*-Ag_2_S at 443 K, the distances Ag1-Ag1 between silver ions are 0.24297 nm, the distance between the nearest Ag1 and Ag2 ions is 0.0943 nm, and spacings Ag2-Ag2 between corresponding nearest silver ions are in the range from 0.6803 to 0.07628 nm. These spacings are noticeably less than the ionic diameter of Ag^+^ ion, equal to ~0.252 nm. Therefore, silver ions in argentite cannot occupy the nearest neighboring sites of the crystal lattice.

**Figure 3:**
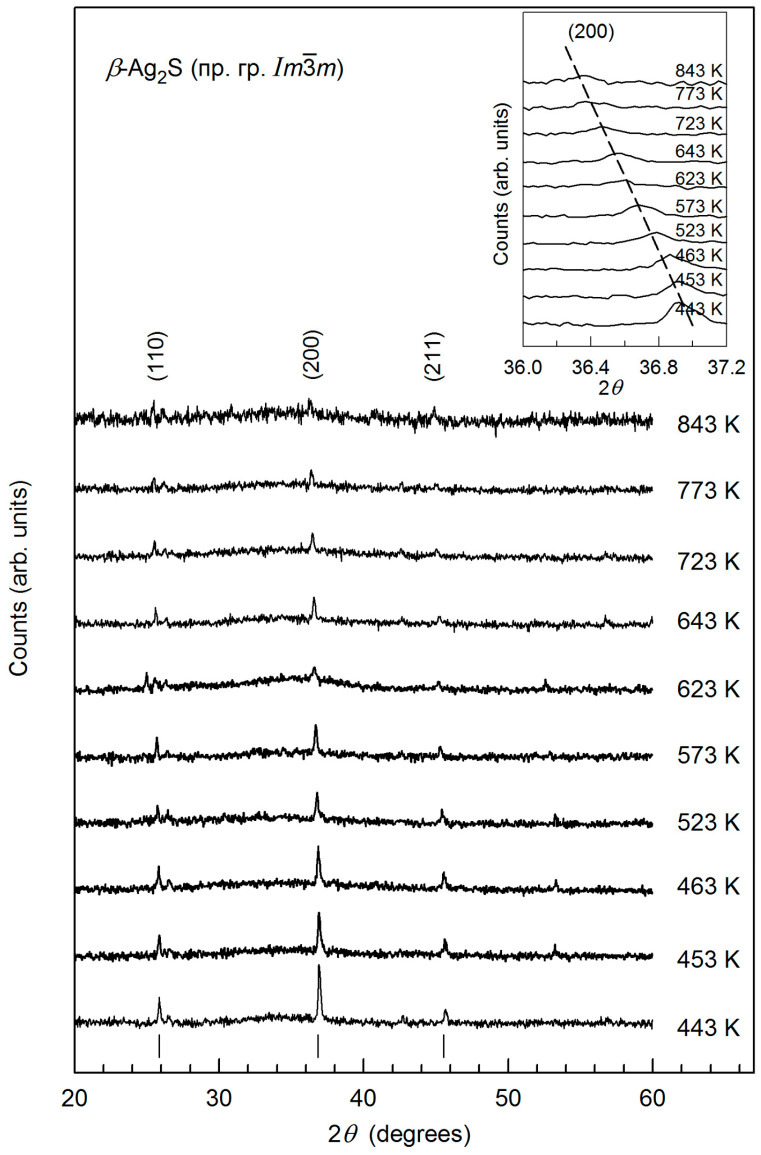
Change of XRD patterns of fine-dispersed argentite *β*-Ag_2_S in the region 443–843 K. As an example, a shift of (200) diffraction reflection with temperature increasing is shown on inset.

**Figure 4:**
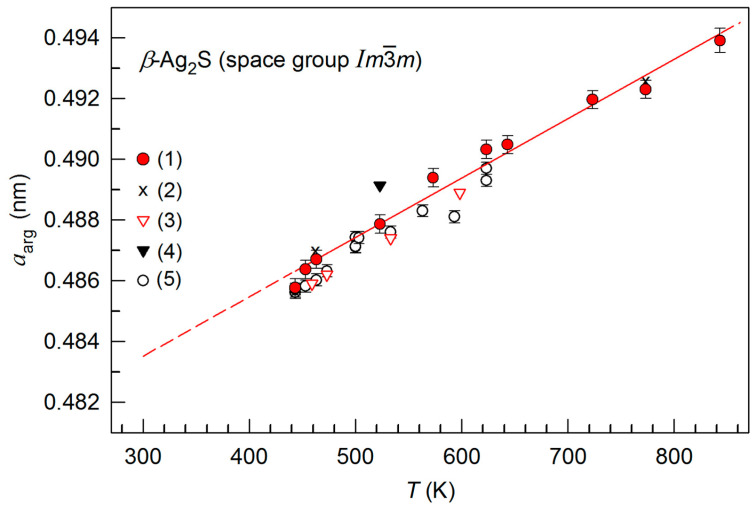
Temperature dependence of the lattice period *a*_arg_ for argentite: (1) present work; (2), (3), (4), and (5) are the data [42,64,65,66], respectively. A solid line depicts the approximation of measured period *a*_arg_ by linear function (2) in the temperature region 443–843 K.

Changes in XRD patterns and parameters of unit cell for *β*-Ag_2_S argentite upon heating from 443 to 843 K are depicted in Figure 3 and Figure 4. The XRD spectra taken during heating from 443 to 843 K include diffraction reflections of only cubic *β*-Ag_2_S argentite (Figure 3). Rise in temperature leads to a continuous shift of the position of the argentite’s reflections to the field of smaller angles 2*θ* (for example, a shift of the (200) diffraction reflection is shown in Figure 3 (inset)). This corresponds to a growth in the lattice parameter *a*_arg_ of argentite upon heating.

The temperature dependence *a*_arg_(*T*) of the lattice period for *β*-Ag_2_S in comparison with the literature data [42,64,65,66] is shown in Figure 4. Within the accuracy of experimental measurements, the dependence of the lattice period *a*_arg_(*T*) in the range 443–853 K is described by a linear function:

*a*_arg_(*T*) = *a*(443) + *b*(*T* − 443), (2)

where *a*(443) = 0.48632 nm and *b* = 1.95684 × 10^−5^ nm·K^−1^. For this linear dependence *a*_arg_(*T*), the coefficient of thermal expansion *α*_arg_ is a constant equal to ~4.024 × 10^−5^ K^−1^. The coefficient *α*_arg_ for bulk argentite at 523 K is 4.58 × 10^−5^ K^−1^ [67]. According to [66], the coefficient *α*_arg_ decreases from ~5.5 × 10^−5^ to ~4.2 × 10^−5^ K^−1^ when the temperature grows from 443 to 623 K. According to the data of direct dilatometric measurements in temperature region 500–800 K, the expansion coefficient *α*_arg_ of *β*-Ag_2_S grows from ~3.02 × 10^−5^ to ~4.21 × 10^−5^ K^−1^ [37,38]. Overall, the coefficient *α*_arg_ of argentite, estimated from the change in the lattice period, is in satisfactory accordance with the available literature data.

### 3.3. γ-Ag_2_S Phase

**Figure 5:**
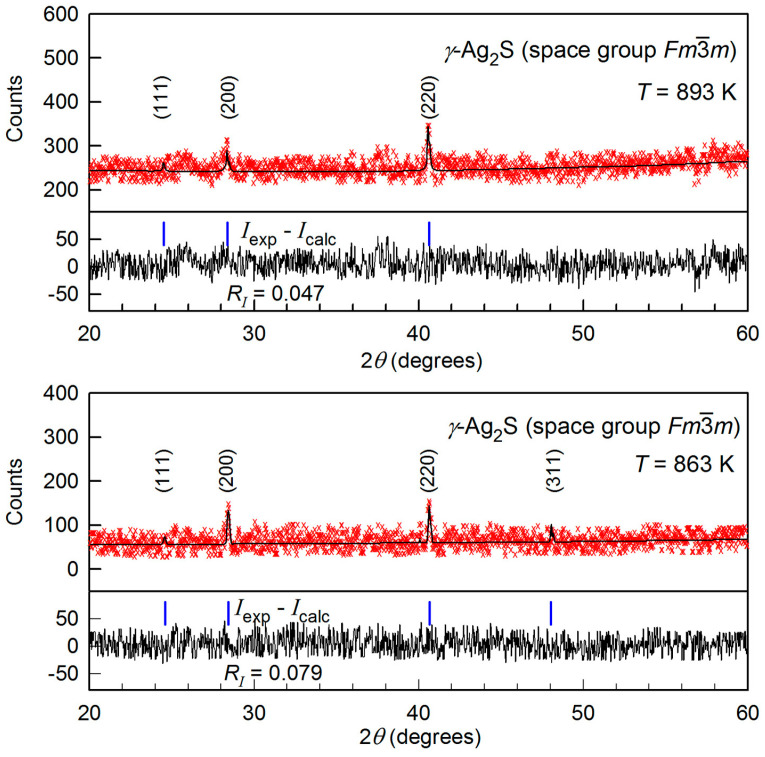
Experimental (**×**), calculated (—), and difference (*I*_exp_—*I*_calc_) XRD patterns of Ag_2_S pressed pill at temperatures of 863 and 893 K. Silver sulfide has a face-centered cubic (fcc) (space group $Fm\bar{3}m$) *γ*-Ag_2_S structure at 863 and 893 K.

The XRD patterns of silver sulfide at 863 and 893 K exhibit reflections of the fcc (space group $Fm\bar{3}m$) *γ*-Ag_2_S phase and broad diffuse bands (Figure 5). The Rietveld convergence factors for refining the structure of Ag_2_S powder at 863 K are *R*_p_ = 20.67%, *ωR*_p_ = 25.52%, *R_I_* (*R*_B_) = 7.973%, and *R*_expect_ = 17.41%. The atomic coordinates and the parameters of fcc unit cell of fine-dispersed powder of silver sulfide at 863 K are presented in Table 3.

From a comparison of the XRD patterns at 843 and 863 K (see Figure 2 and Figure 5) and the data [37,68,69], it follows that the transformation of *β*-Ag_2_S argentite into*γ*-Ag_2_S phase occurs in the temperature range 843–863 K, i.e., at a temperature of ~850–860 K.

**Figure 6:**
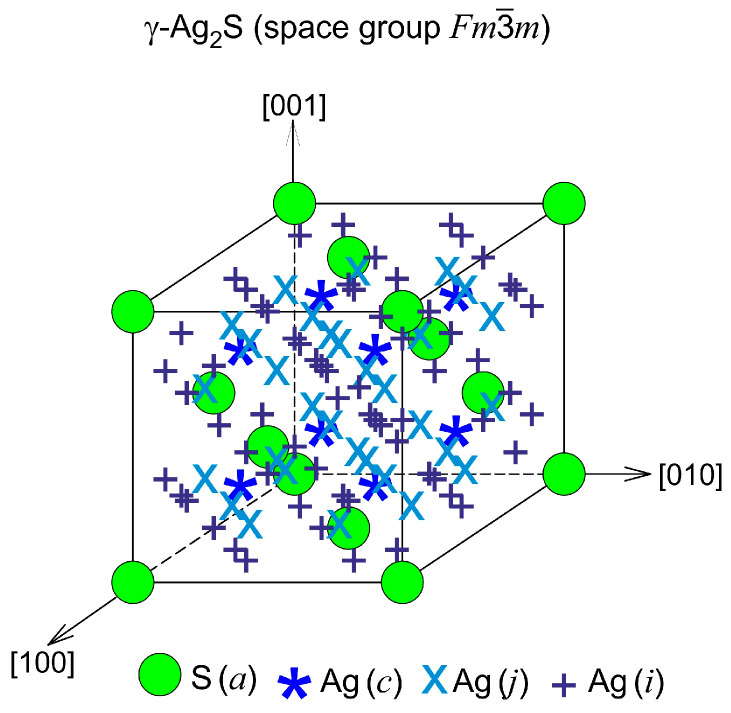
The cubic (space group $Fm\bar{3}m$) unit cell of *γ*-Ag_2_S phase. Exact positions of S atoms and sites of three types, where Ag atoms are located with different probabilities (see Table 3), as shown.

Four Ag_2_S formula units enter into the unit cell of *γ*-Ag_2_S phase (Figure 6). S atoms are in 4(*a*) crystallographic positions. Ag atoms are randomly situated on 88 positions 8(*c*), 32(*j*), and 48(*i*) with the probabilities of filling ~0.088, ~0.150, and ~0.027 (Table 3). Figure 6 presents the exact positions of S atoms and the sites of three types on which the Ag atoms can be located with the mentioned probabilities. According to the refined structure, a noticeable silver deficit corresponding to the nonstoichiometric sulfide Ag_1.7_S is observed in the *γ*-phase. The *γ*-Ag_2_S phase has superionic conductivity, like *β*-Ag_2_S argentite, due to disordered distribution of Ag atoms, low probabilities of filling the lattice sites by Ag atoms, their continuous motion, and silver deficiency. The lattice period of cubic *γ*-Ag_2_S at 863 and 893 K is 0.62706 and 0.62747 nm, respectively.

The calculated interatomic (interstitial) spacings for different coordination spheres (CS) of structure of the fcc (space group $Fm\bar{3}m$) *γ*-Ag_2_S phase are given in Table 4.

Interstitial spacings *d* were calculated using a simple formula for describing structures with a cubic unit cell:

(3) $$d=a\times \sqrt{{\left(x_{1}-x_{2}\right)}^{2}+{\left(y_{1}-y_{2}\right)}^{2}+{\left(z_{1}-z_{2}\right)}^{2}}.$$

For the calculation, we used the coordinates (*x*, *y*, *z*) of atoms in *γ*-Ag_2_S phase (Table 3) and the value of lattice period *a* of *γ*-Ag_2_S phase at 863 K. The occupation probabilities of the sites of metallic sublattice by Ag atoms are small, and therefore it is more correct to speak about the spacings between sites at which the disposition of Ag atoms is possible.

The distances (spacings) between Ag^+^ ions in the γ-Ag2S phase at a temperature of ~860 K were compared with the diameter of Ag^+^ ion at the same temperature. The diameter of Ag^+^ ion for coordination number 6 is ~0.252 nm at 298 K [60]. The linear coefficient of thermal expansion *α*_Ag_ for silver in the temperature region 300–1000 K varies from 19 × 10^−6^ to 25 × 10^−6^ K^−1^ [70], the average coefficient of expansion of silver for this temperature range is *α*_Ag-aver_ ≈ 22 × 10^−6^ K^−1^. Taking this into account, the ionic diameter of silver at ~860–900 K is ~0.255 nm.

At 863 K in the lattice of cubic *γ*-Ag_2_S phase, the possible smallest interionic spacings Ag1-Ag1 are 0.3135 nm, and the distances Ag1-Ag2 between corresponding silver ions are in the region from 0.0909 to 0.2719 nm. Interionic distances Ag2-Ag2 are in the region of 0.1481 to 0.2953 nm, distances between ions Ag2 and Ag3 are in the interval of 0.1076 to 0.2516 nm, and spacing Ag3-Ag3 between corresponding ions lie in the range from 0.0940 to 0.3026 nm. The sites of silver sublattice, especially 48(*i*) sites, are located very close to each other. Therefore, the disposition of the Ag^+^ ion in any site excludes the filling of adjacent site by another silver ion Ag1 or Ag2. Indeed, the diameter of Ag^+^ ion (≥0.255 nm) is more the distances Ag1-Ag3 (0.1637 nm) and Ag2-Ag3 (0.1076–0.2516 nm). The interstitial spacings in *γ*-Ag_2_S phase are such that, in the appearance of Ag^+^ ion at site 8(*c*), another Ag^+^ ion can fill one of the sites 32(*j*), which is only in the 2nd or 3rd CS at a spacing of 0.2712–0.2719 nm from site 8(*c*) (Table 4). The presence of Ag^+^ ion at site 8(*c*) excludes the filling of any site 48(*i*) with Ag3 atom, and vice versa. Similarly, if Ag^+^ ion fills one of the sites 32(*j*), then another Ag^+^ ion can be disposed at site 32(*j*), located at a spacing of leastwise 0.255 nm from the first site, i.e., in the 7th or more distant CS. When the Ag^+^ ion is placed at the site 48(*i*), another Ag^+^ ion can fill one of the 48 sites (*i*) situated in the 5th or more distant CS relative to the first site (see Table 4).

Thus, in cubic *γ*-Ag_2_S phase, the possible spacings between silver atoms (ions) are too small for positions (*c*), (*j*), and (*i*) to be filled by silver ions with a probability equal to 1. Therefore, the degree of occupying positions (*c*), (*j*), and (*i*) by Ag atoms are very small and amount to 0.088, 0.15, and 0.027 (see Table 3).

This means that in the lattice of the fcc *γ*-Ag_2_S phase, as in the lattice of cubic argentite, Ag atoms are in permanent motion along 88 crystallographic positions which are possible for them. Moreover, the very close arrangement of the sites of the silver sublattice in *γ*-Ag_2_S phase leads to a noticeable deviation of its composition from stoichiometry (Ag_1.7_S) with a silver deficiency. Continuous motion of Ag atoms and their deficiency ensures the stability of the fcc *γ*-Ag_2_S phase and its superionic conductivity.

### 3.4. Diffuse Scattering in XRD Patterns of the Superionic β-Ag_2_S and γ-Ag_2_S Phases

**Figure 7:**
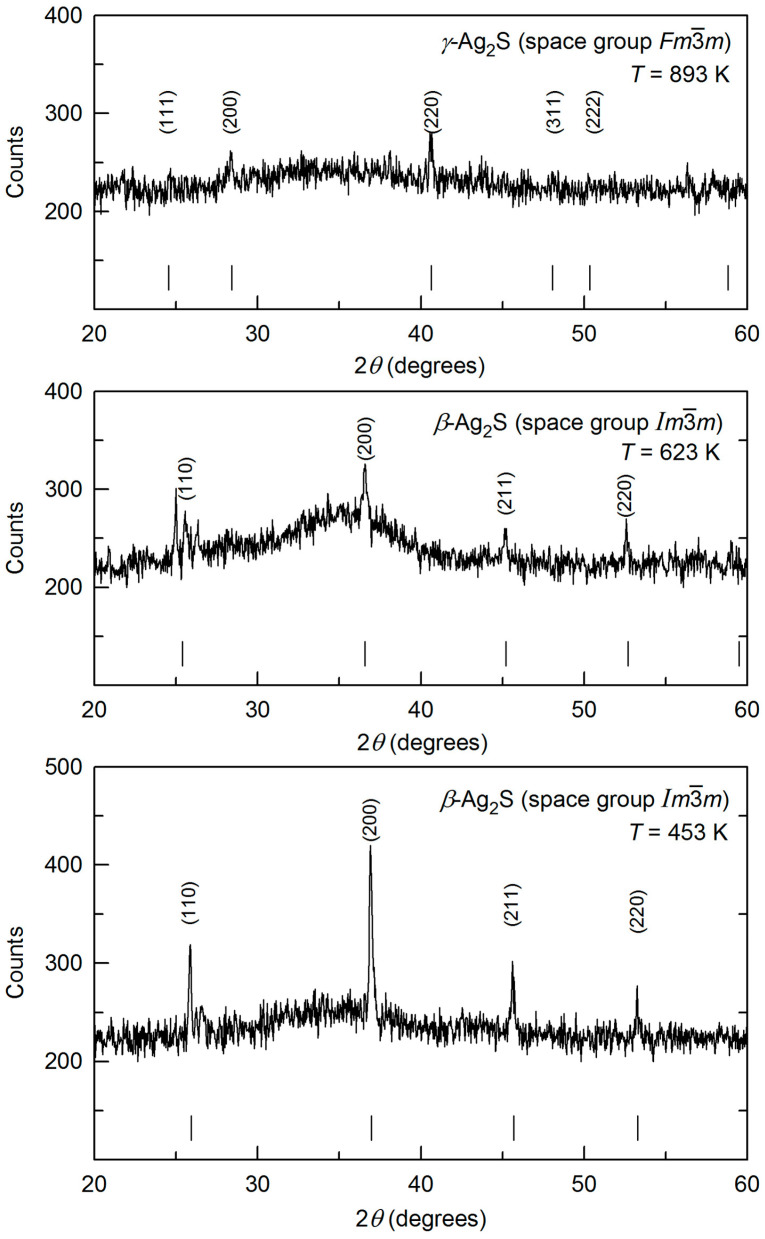
XRD patterns of superionic *β*-Ag_2_S and *γ*-Ag_2_S phases at temperatures of 453, 623, and 893 K.

Regions of strong diffuse scattering are visible in XRD patterns of the superionic *β*-Ag_2_S and *γ*-Ag_2_S phases. Figure 7 shows the XRD patterns of *β*-Ag_2_S argentite at 453 and 623 K and *γ*-Ag_2_S phase at 893 K with regions of strong diffuse scattering.

In XRD patterns of argentite at temperature 453 and 623 K, along with diffraction reflections of cubic *β*-Ag_2_S, diffuse reflection in the region 2*θ* ≈ 30.0–31.0° and a wide diffuse halo in the region 2*θ* ≈ 34–40° are observed (see Figure 7). The intensity of argentite reflections decreases with increasing temperature, while the intensity of the diffuse halo increases. In the XRD pattern of *γ*-Ag_2_S phase at 893 K, there is a wide diffuse halo in the region 2*θ* ≈ 30–39° between the diffraction reflections (200) and (220) of the fcc *γ*-phase.

The appearance of diffuse scattering is due to the very high mobility of Ag atoms in the superionic phases. Earlier, Blanton et al. [42] observed wide diffuse halos in the XRD pattern of argentite at 523 K and in the XRD pattern of *γ*-Ag_2_S phase at 923 K in the same interval of 2*θ* angles. They noted that the weak diffraction of the *β*-Ag_2_S and *γ*-Ag_2_S phases, and the observed peaks of diffuse scattering are due to the large disordering and high mobility of silver atoms.

**Figure 8:**
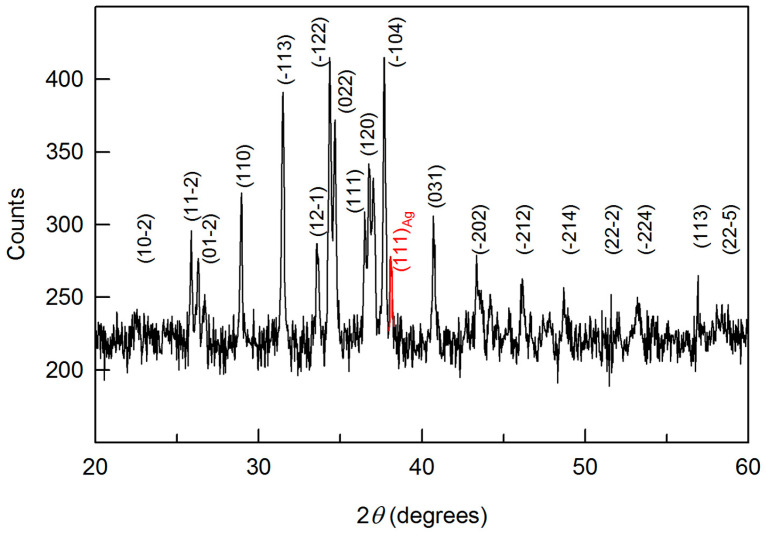
Experimental XRD pattern of Ag_2_S pressed pill registered at 298 K after heating to 893 K. Diffraction reflection (111)_Ag_ of metallic silver Ag is shown by red color.

After heating the sample to 893 K, the XRD pattern (see Figure 7) contains only weak reflections of the fcc *γ*-Ag_2_S phase and a wide diffuse halo. Subsequent cooling of the sample to room temperature restores the monoclinic structure. However, in the XRD pattern, in addition to the reflections of the main monoclinic phase, there is a reflection (111) of cubic (space group $Fm\bar{3}m$) metallic silver. This is clearly seen in Figure 8. Apparently, silver sulfide decomposes at temperatures above 800 K with isolation of metallic silver. Indeed, the composition of the *γ*-phase corresponds to the nonstoichiometric sulfide Ag_1.7_S (see Table 3) due to the precipitation of metallic Ag. The appearance of Ag whiskers on the surface of a pressed pill of silver sulfide heated in air to 570 K was detected in work [71]. The authors of work [42] observed the spontaneous appearance of silver whiskers as a result of heating of Ag_2_S to 800–900 K.

**Figure 9:**
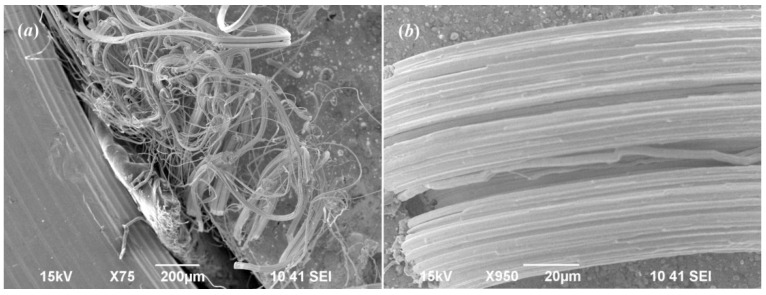
The surface of sample pressed from synthesized fine-dispersed Ag_2_S powder, heated at 893 K and cooled to 298 K: (**a**) long whiskers of metallic silver; (**b**) splices (aggregates) of metallic silver whiskers.

Scanning electron microscopy of a sample pressed from a synthesized fine-dispersed Ag_2_S powder heated to 893 K and cooled to room temperature (Figure 9) confirms the change in the phase composition due to the appearance of Ag whiskers. Numerous long whiskers of metallic silver with a diameter of 1–2 µm appeared on the surface of cooled sample (Figure 9a). In some places of the sintered sample, the whiskers are combined into splices up to 40 μm thick (Figure 9b).

### 3.5. Transitions “α-Ag_2_S–β-Ag_2_S” and “β-Ag_2_S—γ-Ag_2_S” as the First-Order Transformations

**Figure 10:**
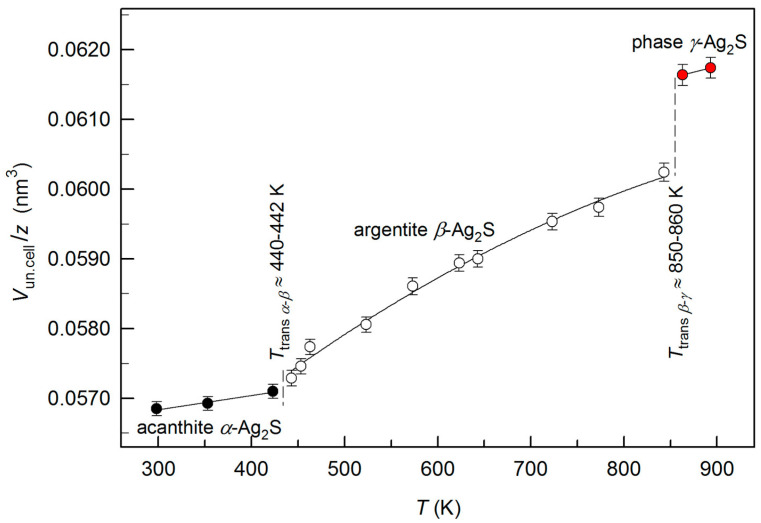
Temperature dependence of reduced volume *V*_un.cell_/*z* of silver sulfide in the region 298–893 K. At ~440–442 K and ~850–860 K, jumps in the reduced volume are observed, which correspond to the first order phase transformations “*α*-Ag_2_S—*β*-Ag_2_S” and “*β*-Ag_2_S—*γ*-Ag_2_S”, respectively.

The *α*-Ag_2_S, *β*-Ag_2_S, and *γ*-Ag_2_S phases, observed at a temperature of 298–893 K, have a different crystal structure. Therefore, to compare the changes occurring in silver sulfide at heating from 298 to 893 K, we used reduced volumes of the unit cells of these phases. The reduced unit cell volume is the volume of unit cell, *V*_un.cell_, divided by the quantity of Ag_2_S formula units, *z*, in the cell, i.e., *V*_un.cell_/*z* (*z* = 4 for acanthite *α*-Ag_2_S and fcc *γ*-Ag_2_S phase, and *z* = 2 for argentite *β*-Ag_2_S). With an increase in temperature from 298 to 893 K, the reduced volume increases and at temperatures of ~420–422 K and ~840–850 K, its abrupt increase is observed. The abrupt changes in the reduced volume are associated with the transformations of acanthite into argentite and argentite into the *γ*-phase of Ag_2_S (Figure 10).

The change in the reduced volume upon the “*α*-Ag_2_S acanthite—*β*-Ag_2_S argentite” transition is noticeably less than that during the “*β*-Ag_2_S argentite—*γ*-Ag_2_S phase” transition. The small value of the jump in the reduced volume at the transition “*α*-Ag_2_S acanthite—*β*-Ag_2_S argentite” is due to the following. According to [57], the structure of *α*-Ag_2_S acanthite is a result of small displacements of S and Ag atoms from positions of bcc lattice of *β*-Ag_2_S argentite. The “acanthite—argentite” transformation occurs due to the jump of Ag^+^ ion from (*e*) acanthite site to (*j*) argentite site [72]. The distance on which Ag^+^ ion is displaced during such jumping is small and amounts to only 0.0425 nm. The energy barrier for the jump of the Ag^+^ ion from the acanthite sites to the argentite sites is from 89 to 410 meV [12,72,73]. During the transition “*β*-Ag_2_S argentite—*γ*-Ag_2_S phase”, the displacements of atoms, especially sulfur atoms S, are larger than at the transition “acanthite-argentite”. As a result, the transition “*β*-Ag_2_S argentite—*γ*-Ag_2_S phase” is accompanied by a larger jump in the reduced volume.

The discontinuous change of the reduced volume during the “acanthite *α*-Ag_2_S—argentite *β*-Ag_2_S” transition corresponds to the transformation of first-order [61,64,69,74]. Measuring the thermal expansion coefficient *α* and the heat capacity *C*_p_ of silver sulfide [37,38] at temperatures from 290 to 970 K also confirms that the “*β*-Ag_2_S—*γ*-Ag_2_S” transition is a first-order phase transformation.

**Figure 11:**
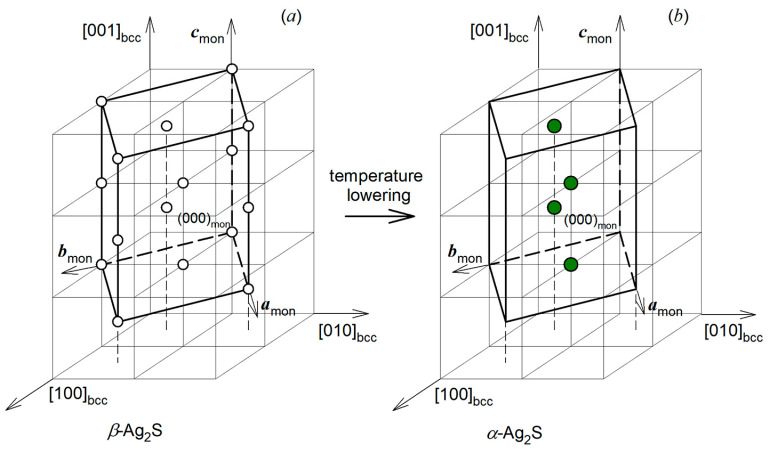
The unit cell of monoclinic acanthite in the bcc nonmetallic sublattice of argentite (only S atoms are depicted). (**a**) Contours of the unit cell of monoclinic acanthite, open circles are S atoms that occupy the sites of argentite with probability equal to 1; (**b**) Simulated monoclinic (space group *P*2/***c***) unit cell of ordered phase *α*-Ag_2_S, closed circles are the S atoms in monoclinic unit cell of ordered phase *α*-Ag_2_S.

An additional theoretical confirmation of the first order of phase transformation “*α*-Ag_2_S acanthite—*β*-Ag_2_S argentite” is the symmetry analysis of this transformation. The transformation of argentite into acanthite, which occurs with a decrease in temperature, can be discussed as a transition from random distribution of Ag atoms in cubic argentite to their ordered arrangement in monoclinic acanthite with a simultaneous change in the positions of sulfur atoms. The location of the monoclinic unit cell in basic bcc nonmetallic sublattice of argentite is shown in Figure 11a, with allowance for the ratio of the axes of acanthite and argentite. When the temperature drops below the transition temperature *T*_trans_, the S atoms, which equally fill the sites of bcc nonmetallic argentite sublattice, concentrate on the 4 sites of monoclinic nonmetallic sublattice (Figure 11b).

**Figure 12:**
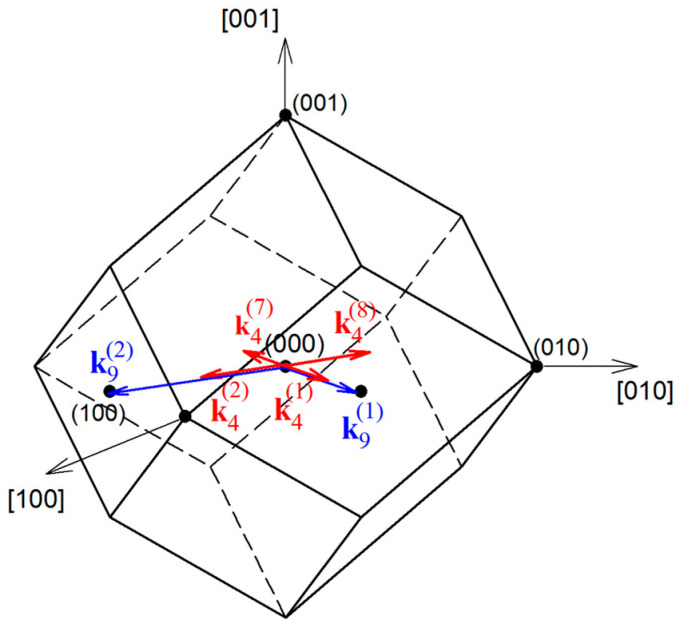
Positions of six superstructural wave vectors $k_{9}^{\left(1\right)}$, $k_{9}^{\left(2\right)}$, $k_{4}^{\left(1\right)}$, $k_{4}^{\left(2\right)}$, $k_{4}^{\left(7\right)}$ and $k_{4}^{\left(8\right)}$ of the simulated monoclinic ordered phase *α*-Ag_2_S in the first Brillouin zone of the bcc lattice.

This makes it possible to discuss the formation of a monoclinic cell as an ordering of S atoms in the bcc sublattice. Four Ag atoms, randomly situated on 54 crystallographic positions of argentite and being in continuous motion due to small distances between these positions, during transition to acanthite find themselves in the sites 4(*e*) with a probability equal to 1. Thus, ordering sulfur atoms in argentite is accompanied by the ordering of silver atoms. The vectors of the reciprocal lattice of the simulated monoclinic *α*-Ag_2_S acanthite are a* = {1 1 0}, b* = {−1/2 −1/2 0}, and c* = {1/4 1/4 1/2} in 2*π*/*a* units (*a* is lattice period of bcc argentite). The combining and translations of these vectors allow calculating an array of superstructural vectors that are situated in the first Brillouin zone of the basic bcc nonmetal sublattice. This array includes six nonequivalent superstructural vectors. Two of them are superstructural wave vectors $k_{9}^{\left(1\right)}$= **b**_3_/2 and $k_{9}^{\left(2\right)}$= (**b**_2_ − **b**_1_)/2 which belong to the 6-ray Lifshitz star {**k**_9_}. Four other superstructural wave vectors $k_{4}^{\left(1\right)}$= ¦*Ì***b**_3_ = (1/4, 1/4, 0) and $k_{4}^{\left(2\right)}$= *μ*(**b**_2_ − **b**_1_) = (1/4, −1/4, 0) and opposite wave vectors $k_{4}^{\left(7\right)}$ = −$k_{4}^{\left(1\right)}$ and $k_{4}^{\left(8\right)}$ = −$k_{4}^{\left(2\right)}$ are the rays of the 12-ray non-Lifshitz star {**k**_4_} with the current parameter *μ*_4_ = 1/4. Here the numbering and the quantitative description of wave vectors stars {**k**_s_} and rays $k_{s}^{\left(j\right)}$ are given according to [75,76]; **b**_1_ = (0 1 1), **b**_2_ = (1 0 1), and **b**_3_ = (1 1 0) are the structure vectors for reciprocal lattice of the basic bcc lattice in 2*π*/*a* units (*a* is the lattice period for the bcc lattice). These six superstructural vectors, $k_{9}^{\left(1\right)}$, $k_{9}^{\left(2\right)}$, $k_{4}^{\left(1\right)}$, $k_{4}^{\left(2\right)}$, $k_{4}^{\left(7\right)}$, and $k_{4}^{\left(8\right)}$, are the phase transition channels associated with the formation of the simulated monoclinic (space group *P*2/*c*) *α*-Ag_2_S ordered phase (Figure 12).

Thus, the “argentite–acanthite” transformation can be discussed as a disorder–order transition in both (nonmetallic and metallic) sublattices of cubic argentite, complicated by atomic static displacements. This transformation occurs with a distortion of cubic symmetry of argentite for the Lifshitz star {**k**_9_} and the non-Lifshitz star {**k**_4_}. The presence of the rays of a non-Lifshitz star in the phase transition channel means that the transformation “*β*-Ag_2_S—*α*-Ag_2_S” does not fulfill the Landau group-theoretical criterion for second-order phase transformations. Therefore, the transformation “*β*-Ag_2_S—*α*-Ag_2_S” occurs on the first-order transition mechanism.

### 3.6. High-Resolution Transmission Electron Microscopy

**Figure 13:**
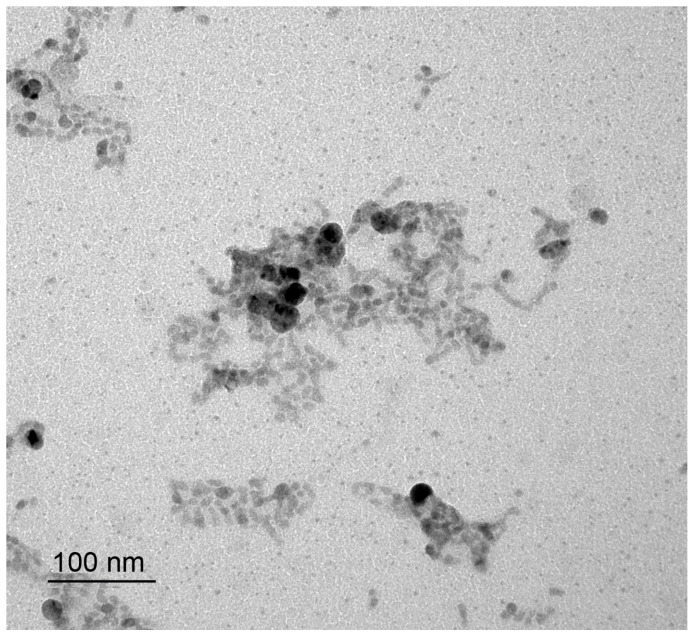
TEM image of synthesized colloid solution of nanoparticles Ag_2_S.

The TEM image of prepared colloid Ag_2_S nanoparticles is presented in Figure 13.

**Figure 14:**
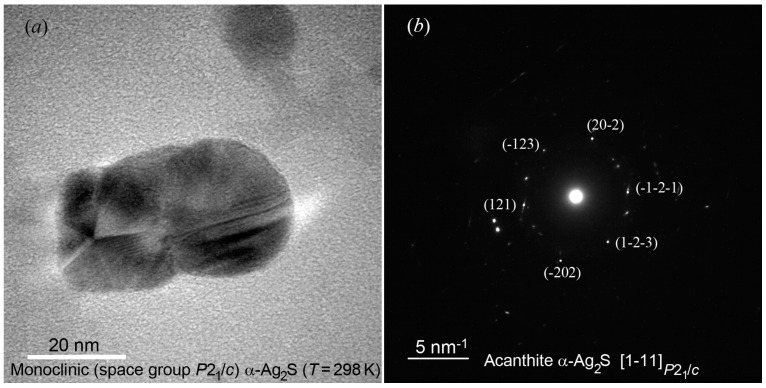
(**a**) HRTEM image of agglomerate of several silver sulfide nanoparticles and (**b**) electron diffraction pattern (the zone axis ${\left[1\text{-}11\right]}_{P2_{1}/c}$) of this image. Electron diffraction pattern contains the reflections corresponding to monoclinic (space group *P*2_1_/*c*) *α*-Ag_2_S acanthite.

Along with separate Ag_2_S nanoparticles of almost the same size 5–6 nm, there are agglomerates −20 nm in size. Agglomerated colloid nanoparticle of silver sulfide at 298 K is presented in Figure 14a. It can be seen that a nanoparticle with a size of ~20–50 nm is agglomerated and combines five or six separate nanoparticles. The electron diffraction of the agglomerated nanoparticle confirms the formation of acanthite. The electron diffraction pattern (Figure 14b) contains diffraction spots of different intensities from several individual monoclinic nanoparticles. In particular, among the observed spots, one can distinguish electron diffraction reflections that belong to monoclinic silver sulfide. The observed set of spots (121), (-123), and (20-2) (Figure 14b) represent the ${\left[1\text{-}11\right]}_{P2_{1}/c}$ plane of the reciprocal lattice of *α*-Ag_2_S acanthite.

**Figure 15:**
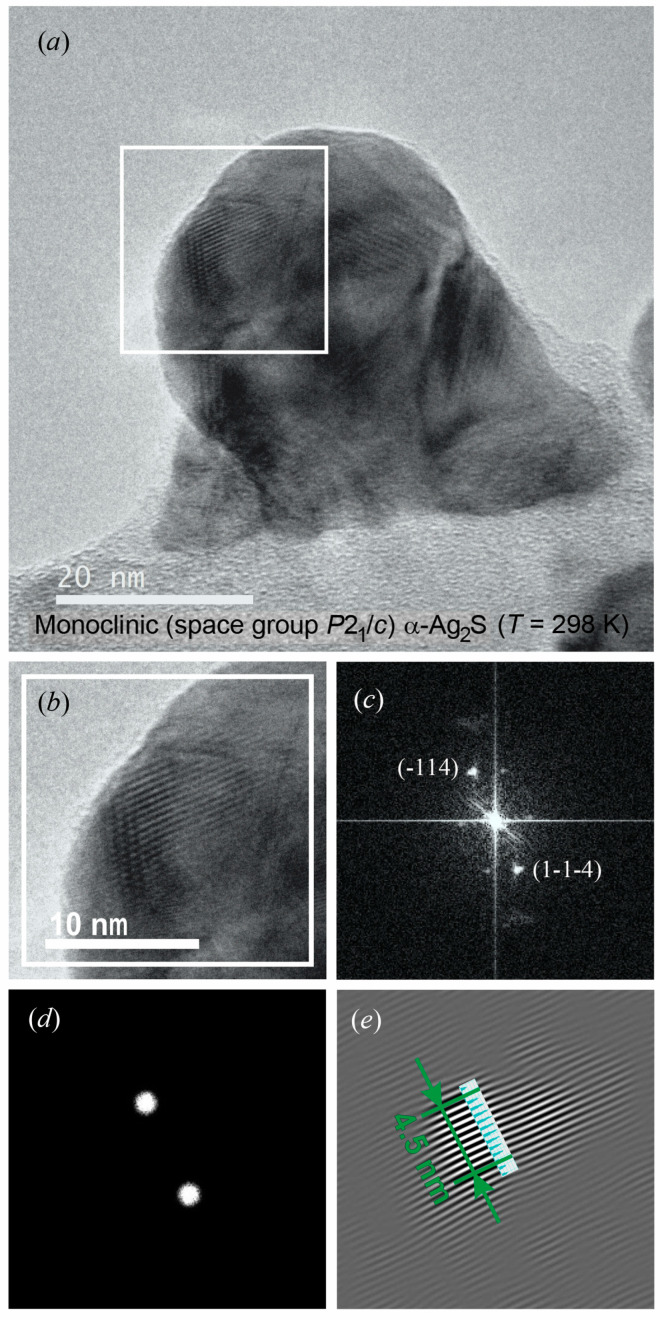
(**a**) HRTEM image of colloid Ag_2_S nanoparticle; (**b**) magnified HRTEM image of the nanoparticle area highlighted by a white square; (**c**) electron diffraction pattern for the same area calculated by the FFT of the magnified HRTEM image; (**d**) a mask created from FFT; (**e**) calculation of inverse FFT with mask using, and generation of line scale of interplanar distances.

Colloid nanoparticle of *α*-Ag_2_S acanthite is shown in Figure 15a. The increased area of the HRTEM image, in which atomic planes are visible, is highlighted with a white square (Figure 15b). The electron diffraction pattern calculated by the Fast Fourier Transform (FFT) of highlighted area of HRTEM image is shown in Figure 15c. To determine the interplanar distance, a mask is created from the FFT image (Figure 15d), which is used to obtain an inverse FFT image of atomic planes and a linear scale of interplanar distances (Figure 15e). Interplanar distances were determined using the DigitalMicrograph software known as the Gatan Microscopy Suite [77]. A detailed description of the determination of interplanar distances using this program [77] is given on the website [78].

The observed distance between ten atomic planes is ~4.504 nm (Figure 15e). The distance between adjacent atomic planes is ~0.450 nm and corresponds to the local ordering of atoms, which leads to the appearance of a superperiodic lattice. A period of a superperiodic lattice is equal to doubled interplanar spacing (−114) of monoclinic acanthite, i.e., 2 × *d*_−114_ ≈ 2 × 0.226 nm. Thus, the ${\left(-114\right)}_{P2_{1}/c}$ atomic planes of monoclinic *α*-Ag_2_S are observed in the HRTEM image (Figure 15a).

**Figure 16:**
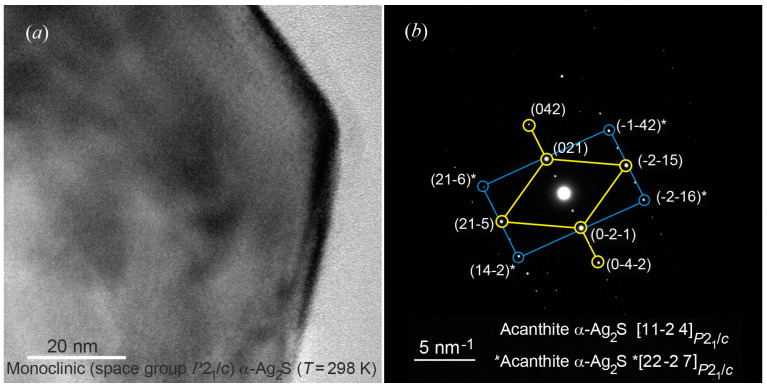
(**a**) HRTEM image of large colloid nanoparticle of *α*-Ag_2_S acanthite in size ~60 nm at 298 K, and (**b**) multiple electron diffraction pattern of this HRTEM image. Electron diffraction pattern (**b**) contains the reflections of different monoclinic *α*-Ag_2_S nanoparticles (the zone axis ${\left[11\text{-}24\right]}_{P2_{1}/c}$ and the zone axis ${*[22\text{-}27]}_{P2_{1}/c}$ ).

HRTEM image of a large colloid nanoparticle of *α*-Ag_2_S acanthite in size ~60 nm, combining several individual nanoparticles, and the observed multiple electron diffraction are shown in Figure 16a,b. The diffraction pattern (Figure 16b) contains diffraction spots of different intensities from several individual monoclinic nanoparticles.

To separate sets of diffraction reflections corresponding to individual nanoparticles, the experimental interplanar distances *d_hkl_* and the angles *φ*_refl_ between (*h*_1_*k*_1_*l*_1_)_mon_ and (*h*_2_*k*_2_*l*_2_)_mon_ reflections were compared with the calculated values of interplanar distances and angles. The interplanar distances and the angles between reflections have been calculated using formulae [6,76]:

(4) $$\frac{1}{d_{hkl}^{2}}={\left(\frac{h}{a\sin\beta }\right)}^{2}+{\left(\frac{k}{b}\right)}^{2}+{\left(\frac{l}{c\sin\beta }\right)}^{2}-2\left(\frac{hl\cos\beta }{ac\sin^{2}\beta }\right),$$

(5) $$\cos\varphi_{\mathrm{refl}}=\frac{h_{1}h_{2}/a^{2}+k_{1}k_{2}/b^{2}+[l_{1}l_{2}a^{2}-(h_{1}l_{2}+h_{2}l_{1})ac\cos\beta +h_{1}h_{2}c^{2}\cos^{2}\beta ]/{\left(ac\sin\beta \right)}^{2}}{d_{1}\times d_{2}},$$

with $d_{i}=\sqrt{{\left(h_{i}/a\right)}^{2}+{\left(k_{i}/b\right)}^{2}+{\left[(l_{i}a-h_{i}c\cos\beta )/(ac\sin\beta )\right]}^{2}}$ where *i* = 1 or 2; *a*, *b*, *c*, *β* are the parameters of unit cell of fine-dispersed monoclinic silver sulfide (see Table 1).

Among the observed diffraction reflections, one can distinguish a set of reflections (021), (042), (0-2-1), (0-4-2), (21-5) and (-2-15), corresponding to the ${\left[11\text{-}24\right]}_{P2_{1}/c}$ plane of the reciprocal lattice of monoclinic *α*-Ag_2_S acanthite. Reflections (21-6)*, (14-2)*, (-2-16)*, (-1-42)* of a monoclinic nanoparticle with diffraction in the ${*[22\text{-}27]}_{P2_{1}/c}$ plane, and some reflections from other nanoparticles are also observed.

### 3.7. Phase Transformations in Silver Sulfide at Temperature from 298 to 893 K

According to the authors of study [79], structural polymorphism and related phase transformations in sulfide nanocrystals are not yet well understood. The research of silver sulfide undertaken fills the noted gap in relation to nanocrystalline Ag_2_S.

**Figure 17:**
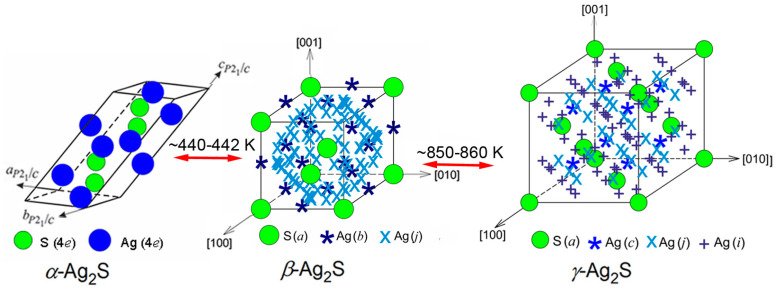
Change of crystal structure of silver sulfide during reversible phase transformations. The unit cells of monoclinic (space group *P*2_1_/*c*) *α*-Ag_2_S acanthite, bcc (space group $Im\bar{3}m$) *β*-Ag_2_S argentite, and fcc (space group $Fm\bar{3}m$ ) *γ*-Ag_2_S phase are presented. The exact positions of S atoms and sites of different types, on which Ag atoms can be located with different probabilities (see Table 2 and Table 3), are shown for the *β*-Ag_2_S argentite and *γ*-Ag_2_S phase.

The results of high-temperature XRD showed that the reversible phase transformations of acanthite to argentite and of argentite to *γ*-Ag_2_S phase take place in the process of heating Ag_2_S silver sulfide. The first transformation occurs at a temperature of ~440–442 K, the second transformation is observed at ~850–860 K (Figure 17). The crystal structures of polymorphic modifications of silver sulfide, especially superionic modifications *β*-Ag_2_S and *γ*-Ag_2_S, are rather complex.

In the *β*-Ag_2_S and *γ*-Ag_2_S phases, Ag atoms are randomly located on several positions of different types with occupancy probabilities less than 1. For *β*-Ag_2_S argentite, there are 54 positions of (*b*) and (*j*) types whose degrees of filling are equal ~0.0974 and ~0.0716. In the *γ*-Ag_2_S, Ag atoms are located at 88 positions of (*c*), (*j*), and (*i*) types with probabilities of filing ~0.088, ~0.150, and ~0.027, respectively. Therefore, the exact positions for S atoms and sites of two and three types are shown for the *β*-Ag_2_S and *γ*-Ag_2_S phases, respectively (Figure 17). Ag atoms can be located at these sites with the indicated probabilities.

The abrupt (discontinuous) change in the reduced volumes of unit cells during the transitions from acanthite to argentite and from argentite to *γ*-Ag_2_S phase indicates that these transitions occur as phase transformations of the first order.

When silver sulfide is heated to a temperature of more than 850 K, the transformation of *β*-Ag_2_S argentite into the *γ*-Ag_2_S phase is accompanied by the isolation of metallic silver. Therefore, the metallic sublattice of the *γ*-phase contains a large number of vacant sites, and *γ*-phase has a nonstoichiometric composition Ag_1.7_S.

## 4. Simulated Arrangements of Ag Atoms in *β*-Ag_2_S Argentite

The unit cells of cubic zinc and silver sulfides *α*-ZnS and *β*-Ag_2_S are shown in Figure S1 (see Supplementary Material).

The low-temperature modification *α*-ZnS has a cubic (space group $F\bar{4}3m$) structure of zinc blende or sphalerite (type *B*3) (Figure S1a) (see Supplementary Material) [80]. The Zn and S atoms occupy the sites of ZnS crystal structure with the probability equaling 1. In contrast to ZnS sphalerite with a fixed arrangement of Zn and S atoms in the unit cell, body-centered cubic (space group $Im\bar{3}m$) *β*-Ag_2_S argentite is characterized by a statistical distribution of Ag atoms [61].

In *β*-Ag_2_S argentite, four Ag atoms are statistically distributed in 54 positions of 6(*b*) and 48(*j*) types (Figure S1b) with degrees of filling ~0.0978 and ~0.0711, respectively [61]. The degrees of filling [61] almost coincide with the found degrees of occupancy (see Table 2). Degrees of filling of 54 positions of argentite silver sublattice are less than 0.1, therefore only sets of 6(*b*) and 48(*j*) positions on which silver ions can be located, are shown on Figure S1b (Supplementary Material). The sites of silver sublattice are so close to each other that the presence of Ag^+^ ion in one of these sites makes the filling of the nearest site by other silver ions impossible because the diameter of Ag^+^ ion is larger than the distance between these sites [81].

To simulate the interface between zinc and silver sulfides, the following should be taken into account. According to [40,53,57,61,72,82], the structure of monoclinic *α*-Ag_2_S acanthite can be considered as result of distortion of body-centered cubic (bcc) sublattice of sulfur atoms S in the structure of cubic *β*-Ag_2_S argentite. At a temperature below 450 K, cubic *β*-Ag_2_S argentite transforms in monoclinic *α*-Ag_2_S acanthite. This transformation is accompanied by a distortion of the bcc sublattice of S atoms to the monoclinic sublattice. Ag atoms that statistically distributed over the sites 6(*b*) and 48(*j*) of the bcc structure of argentite concentrate at the sites of the monoclinic structure of acanthite and occupy them with probability close to one. Thus, at the first stage of simulating the interface, one can consider the interface between cubic zinc sulfide and cubic silver sulfide.

In reality, the distances between Ag atoms in cubic argentite should be larger than the doubled atomic radius of silver, i.e., than ~2.52 Å (0.252 nm). In other words, four Ag atoms in cubic silver sulfide can be located at a distance of at least 2.52 Å from each other. Taking this into account and 48 + 6 = 54 crystallographic positions possible for 4 Ag atoms, there are $C_{4}^{54}$ = 316,251 options for the arrangement of silver atoms in a cubic lattice. In fact, the number of such arrangements is even larger, since it is also necessary to take into account positions in which any coordinate equal to 0 changes to 1. There are 48 + 24 = 72 positions of (*j*) type and 6 + 12 = 18 positions of (*b*) type, i.e., 90 positions for which the number of variants of the arrangement of Ag atoms at arbitrary four positions is $C_{4}^{90}$ = 2,551,190. The coordinates of all positions for the arrangement of Ag and S atoms in the cubic (space group $Im\bar{3}m$) unit cell of *β*-Ag_2_S argentite are given in Table S1 (see Supplementary Material file). Among these arrangements, of interest are those in which the minimum distance between silver atoms exceeds 2.52 Å. The number of such arrangements is 596,898. Most of these options are equivalent and can be obtained from one another by rotating. The number of nonequivalent variants of the arrangement of 4 Ag atoms is much less and is equal to 13,116.

Within the boundaries of the cubic unit cell of argentite, four Ag atoms can be located at four or six positions, depending on the coordinates of these positions. In most cases, silver atoms will not be in the same plane, but there are also such variants of arrangement when all or part of Ag atoms will be in the same plane, or all Ag atoms are in two planes with a certain angle between them.

**Figure 18:**
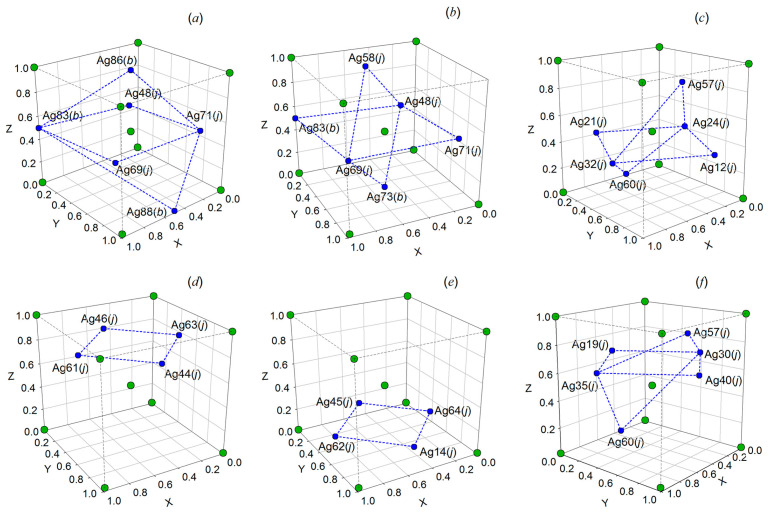
Some of the simulated possible arrangements of four Ag atoms with the location a portion of the Ag atoms in the same plane of the unit cell of cubic *β*-Ag_2_S argentite: (●) metal sites occupied by Ag atoms, (●) nonmetal sites 2 (**a**) occupied by S sulfur atoms.

Such variants of the arrangement of Ag atoms are possible if the following combinations of positions are taken into account: one (*b*) position and three (*j*) positions in the same plane and two (*b*) positions outside this plane (Figure 18a); one (*b*) position and three (*j*) positions in the same plane and one position each (*b*) and (*j*) outside this plane (Figure 18b); four (*j*) positions in the same plane and two (*j*) positions outside this plane (Figure 18c); four (*j*) positions in one plane (Figure 18d,e); four (*j*) positions in the same plane and two (*j*) positions outside this plane (Figure 18f). The arrangement of some Ag atoms in the same plane (z = 0.5) at three (*b*) positions and one (*j*) position is impossible, since in this case the distance between a pair of silver atoms in this plane is less than ~2.52 Å (0.252 nm). Placing Ag atoms in one plane (z = 0.5) only at (*b*) positions is also impossible, since in this case the rest of Ag atoms are located at positions (*b*) and/or (*j*), and the distances between some pairs of silver atoms turn out to be less than ~2.52 Å (0.252 nm). As an example, a few simulated possible arrangements of Ag atoms at different crystallographic positions of the crystal lattice of cubic *β*-Ag_2_S argentite are shown in Figure 18.

Some of the simulated possible arrangements of part Ag atoms at four crystallographic positions located in the same plane of the unit cell of cubic *β*-Ag_2_S argentite are shown in Figures S2–S6 (see Supplementary Material). The coordinates of Ag atoms and interatomic distances between adjacent Ag atoms for each of these possible arrangements are also given there.

## 5. Ag_2_S/ZnS Heteronanostructure

### 5.1. Core-Shell Ag_2_S@ZnS (Ag_2_S/ZnS) Heteronanostructure

**Figure 19:**
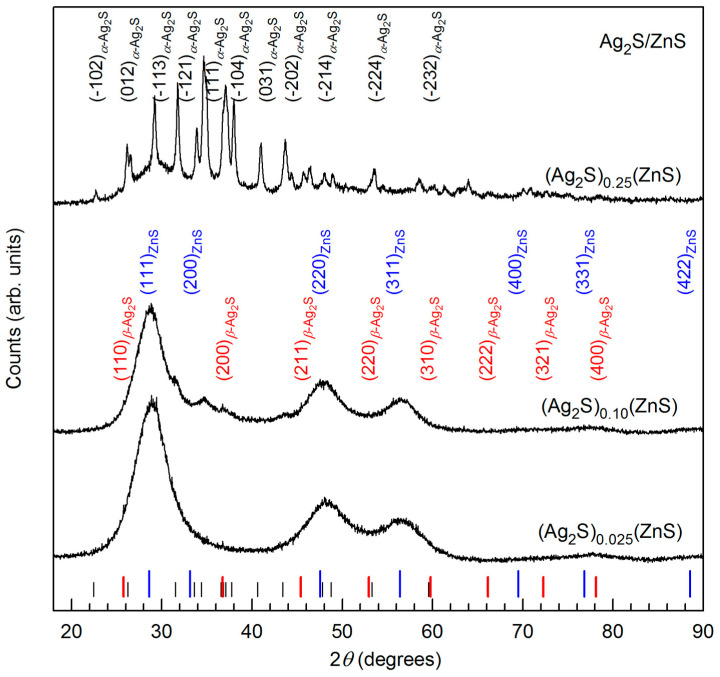
XRD patterns of core-shell Ag_2_S@ZnS (Ag_2_S/ZnS) heteronanostructures: short black, middle red, and long blue ticks indicate reflections from monoclinic (space group P2_1_/c) α-Ag2S acanthite, cubic (space group $Im\bar{3}m$) *β*-Ag_2_S argentite, and cubic (space group $F\bar{4}3m$ ) ZnS sphalerite, respectively.

XRD patterns of synthesized (Ag_2_S)_0.025_(ZnS), (Ag_2_S)_0.1(_ZnS), and (Ag_2_S)_0.25_(ZnS) heteronanostructures are shown in Figure 19. The XRD pattern of the (Ag_2_S)_0.25_(ZnS) heteronanostructure with the highest content of silver sulfide contains diffraction reflections of monoclinic silver sulfide α-Ag_2_S (see Section 3.1) [54,57] and cubic sphalerite ZnS [83]. Quantitative analysis of XRD patterns of (Ag_2_S)_0.1(_ZnS) and (Ag_2_S)_0.025_(ZnS) heteronanostructures with a high content of zinc sulfide and their comparison with the data of Section 3.2 and [42,61,66,83] showed the presence of strong diffraction reflections of cubic sphalerite ZnS and very weak reflections of cubic argentite β-Ag_2_S (Figure 19). The intensity of diffraction reflections of silver sulfide is lower than the intensity of reflections of zinc sulfide due to the low relative content of Ag_2_S in these heteronanostructures. The diffraction reflections of Ag_2_S and, especially, ZnS in heteronanostructures (Ag_2_S)_0.1(_ZnS) and (Ag_2_S)_0.025_(ZnS) are strongly broadened. Broadening of all the reflections of these heteronanostructures is caused by the small size of particles (~9 and ~10 nm for (Ag_2_S)_0.1(_ZnS) and (Ag_2_S)_0.025_(ZnS) heteronanostructures), and the presence of microstrains of crystal lattice owing to its deformation distortions.

Earlier, the formation of Ag_2_S/ZnS heteronanostructures was confirmed in study [84]. According to [84], the central portion of heteronanoparticles ~9–10 nm in size is formed by silver sulfide, and the surface of heteronanoparticles is coated with smaller nanoparticles of cubic zinc sulfide ZnS.

HRTEM confirms the formation of Ag_2_S/ZnS heteronanostructures.

**Figure 20:**
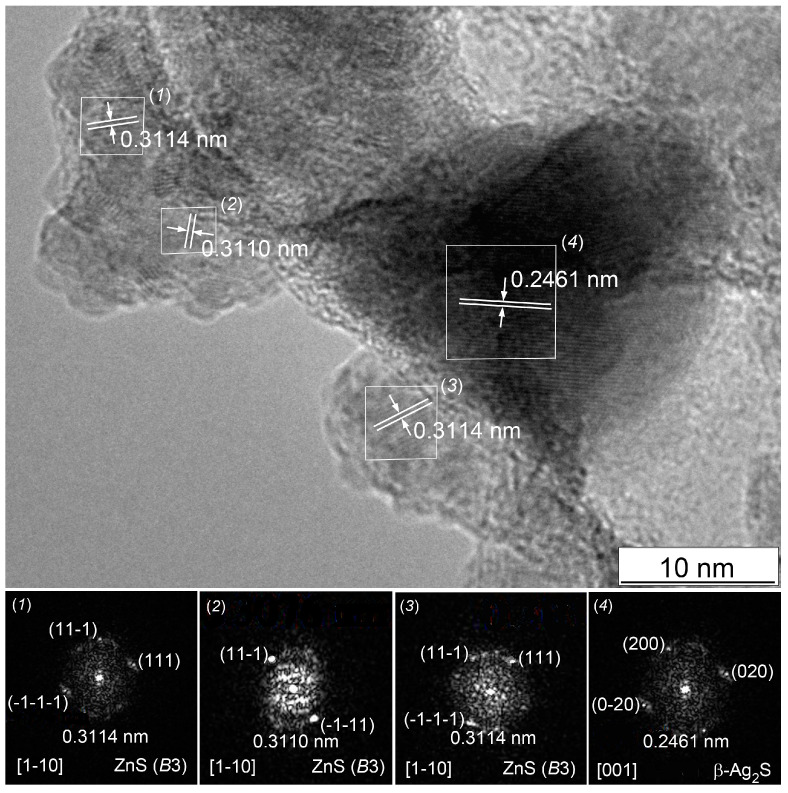
HRTEM image of (Ag_2_S)_0.1_(ZnS) heteronanoparticle. Reprinted with permission from Ref. [84]. 2022, Elsevier. The bottom row shows the electron diffraction patterns obtained by Fast Fourier Transformation (FFT) of selected areas (**1**)–(**4**) of HRTEM image. The interplanar distance of ~0.311 nm for selected areas (**1**)–(**3**) corresponds to the interplanar distance between the atomic planes (111) of cubic (space group $F\bar{4}3m$) ZnS. The interplanar distance ~0.246 nm of selected area (**4**) coincides with the distance between the atomic planes (200) of cubic (space group $Im\bar{3}m$ ) *β*-Ag_2_S silver sulfide.

HRTEM image (Figure 20) confirms the formation of (Ag_2_S)_0.1_(ZnS) heteronanostructure. The dark central part of the heteronanoparticle (selected area (4)) is surrounded by smaller nanoparticles; some of them are selected in areas (1), (2), and (3). The bottom row of Figure 20 shows the electron diffraction patterns obtained by FFT of areas (1)–(4). The set of diffraction reflections and interplanar distances ~0.311 nm (see Figure 20) for areas (1)–(3) correspond to the interplanar distance between the atomic planes (111) of cubic (space group $F\bar{4}3m$) ZnS. The interplanar distance ~0.246 nm of area (4) coincides with the distance between the atomic planes (200) of silver sulfide with a cubic *β*-Ag_2_S argentite structure. Thus, the surface of the core from cubic silver sulfide is covered with a layer of nanoparticles of cubic zinc sulfide ZnS. EDS (EDX) data confirm the presence of Zn, Ag and S in this heteronanostructure (see Figure S7, Supplementary Material). The size of the heteronanoparticle is close to the size estimated from diffraction data. HRTEM image of (Ag_2_S)_0.025_(ZnS) heteronanostructure and its EDS (EDX) data are shown in Figures S8 and S9, respectively (see Supplementary Material). The formation of an interface in the heteronanostructure leads to a change in the band gap E_g_ compared to the band gaps E_g_ of individual sulfides. Such a change in E_g_ from 3.56 eV for (Ag_2_S)_0.025_(ZnS) to 2.06 eV for (Ag_2_S)_0.1_(ZnS) was found in synthesized Ag_2_S/ZnS heteronanostructures with nanoparticles of ~9–10 nm in size [19]. The observed change in the band gap E_g_ additionally confirms the formation in these heteronanostructures of interfaces with minimal strain distortion.

The composition of (Ag_2_S)_y_(ZnS) heteronanostructures can be represented as (Ag_2_S)_1–x_(ZnS)_x_, where x = 1/(1 + y). The obtained experimental diffraction data and TEM results show that (Ag_2_S)_y_(ZnS) heteronanostructures with y > 0.15 (or (Ag_2_S)_1–x_(ZnS)_x_ with x < 0.87), consisting of less than 72 wt. % of cubic zinc sulfide, contain monoclinic silver sulfide. Heteronanostructures (Ag_2_S)**_y_**(ZnS) with y < 0.15 (or (Ag_2_S)_1–x_(ZnS)_x_ with x > 0.87), consist of more than 72 wt. % of cubic zinc sulfide, and contain cubic silver sulfide.

With allowance for the experimental diffraction and TEM data, it can be assumed that a large amount of cubic zinc sulfide (more than 72–73 wt.% ZnS or more than 87 mol.% ZnS) stabilizes the cubic structure of *β*-Ag_2_S argentite at 300 K during the deposition of Ag_2_S/ZnS heteronanostructures from colloid solutions.

### 5.2. Elastic Properties of Cubic Silver and Zinc Sulfides

The elastic stiffness constants c_11_, c_12_, and c_44_ of cubic *β*-Ag_2_S argentite at 470 K and the temperature dependences dc_11_/dT, dc_12_/dT, and dc_44_/dT of these stiffness constants were found in study [40]. According to approximating calculation with allowance for these data, the elastic constants c_11_, c_12_, and c_44_ of cubic *β*-Ag_2_S at 300 K are equal to 99.4, 7.6, and 19.0 GPa, respectively.

For cubic (space group $F\bar{4}3m$) ZnS with a B3 type structure, the data [23,27] are the closest to the elastic properties of cubic Ag_2_S. According to [27], the elastic stiffness constants of cubic ZnS sphalerite at T = 0 K are c_11_ = 96.9, c_12_ = 48.3, and c_44_ = 55.8 GPa.

The values of elastic stiffness constants c_ij_ of zinc sulfide ZnS with cubic (space group $F\bar{4}3m$) sphalerite structure at a temperature of 300 K were estimated using the temperature dependences of the isothermal bulk modulus B presented in studies [31,32]. According to [31], the slope dB/dT is equal to −0.0109 GPa K^−1^. As a first approximation, we will assume that the relative decrease of elastic stiffness constant c_11_ with an increase in temperature from 0 to 300 K is the same as the decrease in the bulk modulus B. The elastic stiffness constants c_ij_ of ZnS at a temperature of 300 K can be represented as c_ij_(T) = c_ij_(0) + Tdc_ij_/dT. According to estimation performed for ZnS, the values of dc_11_/dT, dc_12_/dT, and dc_44_/dT are equal to −0.0109, −0.0057, and −0.022 GPa K^−1^, respectively. The elastic stiffness constants of cubic ZnS sphalerite at a temperature of 300 K are c_11_ = 93.6, c_12_ = 46.6, and c_44_ = 49.2 GPa.

The relationships between stiffness constants c_11_, c_12_, c_44_, and compliance constants s_11_, s_12_, s_44_ for cubic crystals [85] have the simple form: s_11_ = (c_11_ + c_12_)/[(c_11_ − c_12_)(c_11_ + 2c_12_)], s_12_ = −c_12_/[(c_11_ − c_12_)(c_11_ + 2c_12_)], and s_44_ = 1/c_44_. Accordingly, at 300 K, s_11_ = 10.17 × 10^−12^, s_12_ = −0.72 × 10^−12^, and s_44_ = 52.63 × 10^−12^ Pa^−1^ for *β*-Ag_2_S argentite. Similarly, at 300 K the components of the compliance tensor for ZnS sphalerite are s_11_ = 15.97 × 10^−12^, s_12_ = −5.31 × 10^−12^, and s_44_ = 20.33 × 10^−12^ Pa^−1^.

### 5.3. Anisotropy of Elastic Properties

According to [40,48,85,86], the values of the Young’s modulus E, the shear modulus G, and the Poisson’s ratio *μ* of cubic monocrystals depend a direction [*hkl*] through parameter Γ = (*h*^2^*k*^2^ + *h*^2^*l*^2^ + *k*^2^*l*^2^)/(*h*^2^ + *k*^2^ + *l*^2^) as

(6) $$E_{hkl}=\frac{1}{s_{11}-2(s_{11}-s_{12}-\frac{1}{2}s_{44})\Gamma },$$

*Ghkl* = 1/[2s11 − 2s12 − 6(s11 − s12 − s44/2)Γ], (7)

(8) $$\mu_{hkl}=\frac{1-E_{hkl}(s_{11}+2s_{12})}{2}.$$

The bulk modulus B of cubic crystals equals B = 1/[3(s_11_ + 2s_12_)].

The distributions of elastic characteristics of monocrystalline particles of cubic ZnS sphalerite and cubic *β*-Ag_2_S argentite from the [hkl] direction were calculated using the obtained data on s_11_, s_12_, and s_44_ of these sulfides. The dependences of the elastic properties of cubic ZnS and cubic *β*-Ag_2_S from the [hkl] direction are shown in Figure S10 (Supplementary Material).

**Figure 21:**
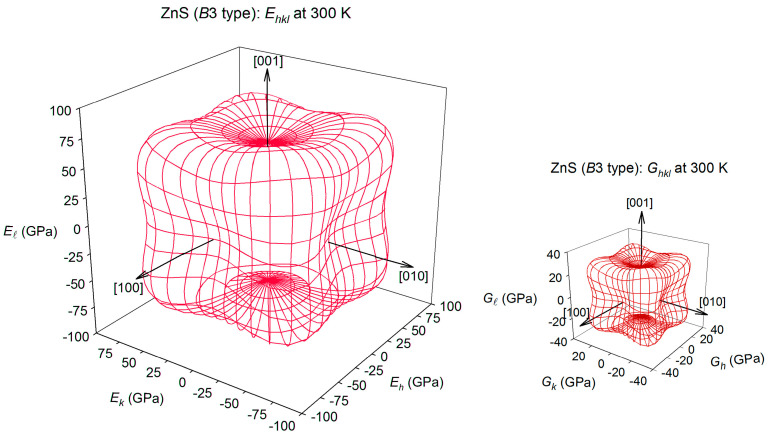
The spatial distributions of the Young’s modulus *E_hkl_* and shear modulus *G_hkl_* for cubic zinc sulfide (ZnS sphalerite) at 300 K.

**Figure 22:**
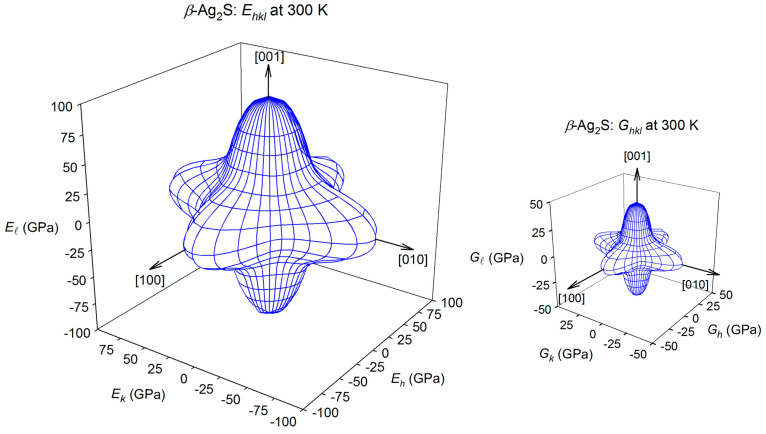
The spatial distributions of the Young’s modulus *E_hkl_* and shear modulus *G_hkl_* for cubic *β*-Ag_2_S argentite at 300 K.

The spatial distributions of the Young’s moduli E_hkl_ and shear modulus G_hkl_ for cubic ZnS sphalerite and cubic *β*-Ag_2_S argentite at 300 K are presented in Figure 21 and Figure 22.

The spatial distribution of the Young’s modulus E_hkl_ of cubic ZnS sphalerite at 300 K is shown in Figure 21. According to the E_hkl_ surface visualization, the Young’s modulus E_hkl_ of cubic ZnS has the maximal value ~116.6 GPa in eight equivalent directions [±1±1±0.6]. At 300 K, the minimal Young’s modulus value ~62.5 GPa of cubic ZnS is observed in directions [100], [010], and [001], and opposite directions. The shear modulus G_hkl_ of sphalerite in the (100) plane has a maximum value of ~38.6 GPa in the [0±1±1] directions. The minimum shear modulus ~23.5 GPa corresponds to the [00±1] and [0±10] directions (Figure 21).

For cubic *β*-Ag_2_S argentite at 300 K, the Young’s modulus E_hkl_ has the maximal value in directions [100], [010], and [001], and opposite directions. At 300 K, the maximal Young’s modulus E_hkl_ is equal to ~98.3 GPa, and the minimal value of ~48.9 GPa is observed for the Young’s modulus E_hkl_ of argentite *β*-Ag_2_S in eight equivalent directions [±1±1±0.6]. The shear modulus G_hkl_ of argentite in the (100) plane has a maximum value of ~44.9 GPa in the [001], [010], and [100] directions and in the opposite directions. The minimal value of ~19.0 GPa is observed for the shear modulus G_hkl_ of argentite *β*-Ag_2_S in eight equivalent directions [±1±1±0.6]. In the (100) plane, the minimum shear modulus ~22.3 GPa corresponds to the directions [0±1±1] (Figure 22).

### 5.4. Interface between Cubic ZnS and Ag_2_S with Simulation of Ag Atoms Arrangement

According to the Hartman–Perdok theory [50], for the considered cubic zinc and silver sulfides, the (111) planes should coincide at the interface. The average shear moduli G_hkl_ of sphalerite ZnS and argentite *β*-Ag_2_S in the (111) planes are ~41.6 and ~21.2 GPa, respectively. In the case of the formation of an interface along the (111) planes of sphalerite and argentite, the difference in their shear moduli G_hkl_ is very large, and the elastic strain energy E_str_ of the (A_g_2S)_0.09_(ZnS)_0.91_ heteronanostructure is large and amounts to 9680 J·mol^−1^ [49]. Therefore, the morphological approach [50] used in study [49], according to which the (111) planes of sphalerite and argentite can be combined at the interface in the Ag_2_S/ZnS heteronanostructure, gives an incorrect result.

In Section 4 and Supplementary Material file, it is shown that among the nonequivalent variants of the arrangement of Ag atoms with distances between them not less than ~2.52 Å (0.252 nm), there are the arrangements of silver atoms at four crystallographic positions located in one plane of the unit cell of cubic *β*-Ag_2_S. The crystallographic positions of these arrangements have the same coordinate z ≡ l, and the z value (taking into account coordinates of Ag atoms (see Table S1, Supplementary Material)) for such arrangements is 0.1694, 0.3306, 0.4123, 0.5, 0.6694, or 0.8306. The distances between the nearest neighboring Zn atoms in the (110) plane of ZnS sphalerite are 0.383 nm. The distances between the nearest neighboring Ag atoms in the (11l) planes with *l* = const of *β*-Ag_2_S argentite depend on the value of l and are equal to 0.363 nm at *l* = 0.4213. At l = 0.5, interatomic Ag-Ag distances are 0.364 and 0.406 nm. At *l* = 0.6694, interatomic Ag-Ag distances are equal to 0.349 nm. Thus, the distances between the nearest neighboring Zn atoms in the (110) plane of ZnS sphalerite, on the one hand, and between the nearest neighboring Ag atoms in the (11l) planes with *l* = const of *β*-Ag_2_S argentite, on the other hand, differ by no more than ~5.2% at *l* = 0.4213, by ~4.7–6.0% at *l* = 0.5, and by ~8.9% at *l* = 0.6694. Since the faces (110) of sphalerite and (11l) with *l* = constant of argentite are characterized by rather close distances between metal atoms, the formation by these faces of the interfaces in Ag_2_S/ZnS heteronanostructures is quite possible. An even more important parameter determining the possibility of forming an interface in Ag_2_S/ZnS heteronanostructure is the smallest strain distortion at the interface between two sulfides.

**Figure 23:**
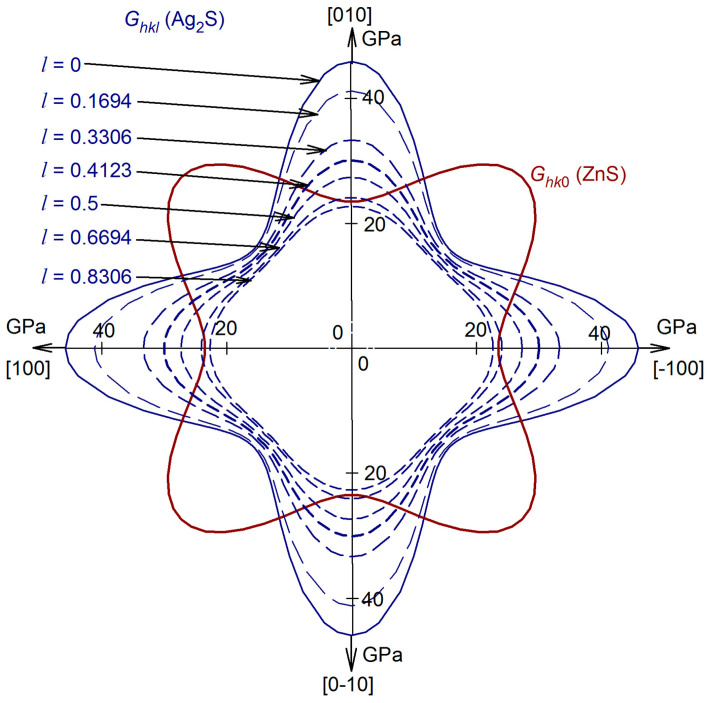
Comparison of the shear moduli G_hkl_ of cubic *β*-Ag_2_S argentite in the (hkl) planes with *l* = 0, 0.1694, 0.3306, 0.4123, 0.5, 0.6694, and 0.8305 with the shear modulus G_hk0_ of cubic ZnS sphalerite in the (hk0) plane. Reprinted with permission from Ref. [84]. 2022, Elsevier.

The distributions of the shear modulus G_hkl_ of argentite in the (*hkl*) planes with *l* = 0.1694, 0.3306, 0.4123, 0.5, 0.6694, or 0.8306 calculated in study [84] are shown in Figure 23. The maximum shear modulus G_hkl_ in the (hkl) planes with l = 0.1694, 0.3306, 0.4123, 0.5, 0.6694, or 0.8306 takes in the [±10 l] and [0±1 l] directions, and the minimum shear modulus G_hkl_ of *β*-Ag_2_S argentite is observed in four equivalent directions [±1±1 l]. As the value of l increases, the shear modulus of *β*-Ag_2_S generally decreases. For comparison, a calculated distribution of the shear modulus G_hk0_ of cubic ZnS sphalerite is shown in Figure 23.

The smallest strain distortions at the interface between silver and zinc sulfides in Ag_2_S/ZnS heterostructures will be observed with a minimum difference in shear moduli of *β*-Ag_2_S and ZnS sulfides. In cubic *β*-Ag_2_S argentite, there are 13,116 nonequivalent variants of the arrangement of 4 Ag atoms (see Section 4). For the formation of Ag_2_S/ZnS heterostructures, such arrangements of Ag atoms in *β*-Ag_2_S argentite are most favorable when all four Ag atoms are in one plane. For the binary or pseudobinary systems, the lowest elastic strain energy is observed when the difference in shear moduli of the system’s components is minimal [87]. Thus, the energetically most favorable will be the Ag_2_S/ZnS heterostructures, with the minimum difference in the shear moduli of the *β*-Ag_2_S and ZnS sulfides.

In accordance with study [87], the elastic strain energy *E_str_* as the function of the (Ag_2_S)_1–x_(ZnS)_x_ composition can be represented as

(9) $$E_{\mathrm{str}}=2x(1-x){\left(V_{{\mathrm{Ag}}_{2}S}-V_{\mathrm{ZnS}}\right)}^{2}[(1-x)G_{{\mathrm{Ag}}_{2}S}/V_{{\mathrm{Ag}}_{2}S}+xG_{\mathrm{ZnS}}/V_{\mathrm{ZnS}}]/3$$

where $G_{{\mathrm{Ag}}_{2}S}$ and *G*_ZnS_ are the shear moduli of Ag_2_S and ZnS sulfides; $V_{{\mathrm{Ag}}_{2}S}$ = 3.43 × 10^−5^ m^3^·mol^−1^ and *V*_ZnS_ = 2.39 × 10^−5^ m^3^·mol^−1^ are the molar volumes of Ag_2_S and ZnS sulfides; x is the relative content of zinc sulfide ZnS in the (Ag_2_S)_1-x_(ZnS)_x_ heteronanostructure. For (Ag_2_S)_0.1_(ZnS) and (Ag_2_S)_0.025_(ZnS) heteronanostructures, the *x* value is ~0.91 and ~0.95, respectively.

The shear moduli that correspond to (hk 0.4123) and (hk 0.5) planes of argentite *β*-Ag_2_S is closest to the shear modulus G_hk0_ of sphalerite ZnS (see Figure 22). The average shear modulus G_hk0_ of ZnS sphalerite is ~30.5 GPa, and the average shear modulus in the (hk 0.4123) and (hk 0.5) planes of *β*-Ag_2_S argentite is ~24.9 and ~23.4 GPa, respectively. Thus, the Ag_2_S/ZnS heterostructures, in which the interface is formed by the (hk0) ≡ (110) plane of ZnS sphalerite and the (hk 0.4123) ≡ (1 1 0.4123) plane of *β*-Ag_2_S argentite, are the most energetically favorable.

The elastic strain energies *E*_str_ of (Ag_2_S)_0.1_(ZnS) and (Ag_2_S)_0.025_(ZnS) heteronanostructures, calculated with allowance for the average shear moduli of sphalerite and argentite *G*_ZnS_(hk0) = 30.5 GPa and $G_{{\mathrm{Ag}}_{2}S(hk\;0.4123)}$ = 24.9, are equal to 7240 and 4250 J·mol^−1^, respectively.

In the first approximation, the interfacial energy γ can be estimated as γ = *E*_str_/S, where S is the surface area of the interface. The surface area of the interface can be found as the average value S = (*V*_ZnS_/*D*_ZnS_ + $V_{{\mathrm{Ag}}_{2}S}/D_{{\mathrm{Ag}}_{2}S}$)/2 where *D*_ZnS_ and $D_{{\mathrm{Ag}}_{2}S}$ is the size of ZnS and Ag_2_S nanoparticles. According to TEM data, the size of nanoparticles of zinc and silver sulfides is the same approximately, and equal to ~10 nm (see Figure 20 and Figure S9), therefore S = 2910 m^2^·mol^−1^. The energies γ of the Ag_2_S(1 1 0.4123)/ZnS(110) interface for the (Ag_2_S)_0.1_(ZnS) and (Ag_2_S)_0.025_(ZnS) heteronanostructures at this S value are ~2.5 and ~1.5 J·m^−2^, respectively.

Knowledge of the elastic stiffness constants of a monocrystal allows one to find the elastic characteristics of a polycrystal using the Voigt–Reuss–Hill method [88].

The bulk B and shear G moduli are related to the elastic stiffness constants c_ij_ and the elastic compliance constants s_ij_ (see Section S2, Supplementary Material). For any crystal system, the bulk and shear moduli are given by the known equations of Voigt [89] and Reuss [90] where B_V_ and B_R_ are the upper and lower limits of bulk modulus B, and *G*_V_ and *G*_R_ are the upper and lower limits of shear modulus *G*, respectively.

According to the Voigt–Reuss–Hill averaging method [88], for cubic crystals:

*B*_VRH_ = (*B*_V_ + *B*_R_)/2 = *B*_V_ = *B*_R_, (10)

*G*_VRH_*= (G*_V_ + *G*_R_)/2, (11)

where *B*_VRH_ and *G*_VRH_ are the isotropic bulk and shear moduli, i.e., the Voigt–Reuss–Hill averages of the bulk and shear moduli, respectively. The isotropic Young’s modulus E and the isotropic Poisson ratio µ are equal to

*E* = (9*B*_VRH_·*G*_VRH_)/(3*B*_VRH_ + *G*_VRH_),   *μ* = (1/2)[(3*B*_VRH_ − *E*)/3*B*_VRH_]. (12)

The Voigt–Reuss–Hill averages of the bulk and shear moduli, as well as estimated isotropic for the Young’s modulus E and the Poisson’s ratio µ for cubic β-Ag_2_S and ZnS sulfides, are given in Table 5. Universal criterion A^U^ = (5*G*_V_/*G*_R_+*B*_V_/*B*_R_-6) is used to quantify the anisotropy of elastic properties of crystals [91]. The value A^U^ = 0 shows the isotropy of crystal. The higher the value of A^U^ is, the more intense the elastic anisotropy is. We used the criterion A^U^ for cubic *β*-Ag_2_S argentite and ZnS sphalerite to evaluate their elastic anisotropy. For the temperature 300 K, the anisotropy criterions A^U^ are 1.008 and 0.687 for cubic *β*-Ag_2_S argentite and ZnS sphalerite (Table 5). Thus, polycrystalline *β*-Ag_2_S argentite and ZnS sphalerite possess the anisotropy of elastic properties.

## 6. Conclusions

Polymorphism, being the ability of certain substances to exist in states with different crystal structures, is quite common among sulfides. Silver sulfide Ag_2_S has three polymorphic modifications. Literature data on polymorphic modifications of Ag_2_S refer mainly to bulk (coarse-crystalline) silver sulfide.

In this work, we have systematically studied the structure of polymorphic modifications of nanocrystalline silver sulfide with a particle 90 nm in size and less. The structure of the superionic *β*-Ag_2_S and *γ*-Ag_2_S phases has been studied in particular detail. It is shown that the interstitial distances between silver ions in the *β*-Ag_2_S and *γ*-Ag_2_S phases are noticeably smaller than the diameter of the Ag^+^ ion, as a result of which the probabilities of filling the sites of the metallic sublattices of these phases with Ag atoms are very small. As a result, silver atoms are in permanent motion over all the crystallographic positions, which is possible for them. Permanent motion of silver atoms creates the superionic conductivity of the *β*-Ag_2_S and *γ*-Ag_2_S phases, and offers the stability of these phases.

It is established that the “*α*-Ag_2_S—*β*-Ag_2_S” и “*β*-Ag_2_S—*γ*-Ag_2_S” transitions between polymorphic modifications occur by the mechanism of first-order transformation at temperatures of ~440–442 K and ~850–860 K, respectively.

In this work, we succeeded in obtaining a core/shell heteronanostructure in which the core of cubic superionic *β*-Ag_2_S argentite is covered with a layer of cubic zinc sulfide ZnS nanoparticles. Ag_2_S/ZnS heteronanostructures were prepared via a facile two-stage hydrochemical co-deposition of Ag_2_S and ZnS from aqueous solutions. Deposition of ZnS on a surface of Ag_2_S nanoparticles has led to the formation of Ag_2_S/ZnS heteronanostructures.

Experimental diffraction and TEM data allow supposing that a large amount of cubic zinc sulfide (more than 87 mol.% ZnS) stabilizes the cubic structure of *β*-Ag_2_S argentite at 300 K during the deposition of Ag_2_S/ZnS heteronanostructures from colloid solutions.

The physically possible variants of the arrangement of silver atoms with a minimum interatomic Ag-Ag distance of more than 2.52 Å on fixed crystallographic positions of cubic argentite are determined.

The elastic stiffness constants *c*_11_, *c*_12_, and *c*_44_ of cubic *β*-Ag_2_S argentite and ZnS sphalerite at a temperature of 300 K are estimated.

It is shown that the formation of Ag_2_S/ZnS heteronanostructures, in which the interface is formed by the (*hk*0) plane of ZnS sphalerite and the (*hk* 0.4123) plane of *β*-Ag_2_S argentite, is the most energetically favorable. The smallest strain distortions at this interface are observed. The interfacial energy of the Ag_2_S/ZnS heteronanostructures is estimated.

Calculation of universal criterion of the anisotropy of elastic properties of cubic *β*-Ag_2_S argentite and ZnS sphalerite shows that the polycrystalline cubic silver and zinc sulfides are elastically anisotropic.

## Figures and Tables

**Table 1:** Refined crystal structure of synthesized silver sulfide with monoclinic (space group *P*2_1_/*c*) *α*-Ag_2_S acanthite structure at 298 K: *Z* = 4, *a* = 0.42294(4) nm, *b* = 0.69324(7) nm, *c* = 0.95352(8) nm, and *β* = 125.566(3)°.

Atom	Position and Ultiplicity	Coordinates of Atoms			Occupancy	*B*_iso_ × 10^−4^ (pm^2^)
		*x*	*y*	*z*		
Ag1	4(*e*)	0.07118	0.01689	0.30750	1.00	9.45(8)
Ag2	4(*e*)	0.72588	0.32128	0.43619	1.00	9.25(7)
S	4(*e*)	0.50000	0.23826	0.13060	1.00	0.50

**Table 2:** Refined crystal structure of bcc (space group $Im\bar{3}m$ ($O_{h}^{9}$)) fine-dispersed powder of silver sulfide with *β*-Ag_2_S argentite structure at 443 K: *Z* = 2, *a* = *b* = *c* = 0.4859(3) nm.

Atom	Position and Multiplicity	Coordinates of Atoms			Occupancy	*B*_iso_ × 10^−4^ (pm^2^)
		*x*	*y*	*z*		
Ag1	6(*b*)	0	0.5	0.5	0.09740	0.50
Ag2	48(*j*)	0	0.3190	0.4300	0.07159	0.50
S	2(*a*)	0	0	0	1.00	0.50

**Table 3:** Refined crystal structure of cubic (space group $Fm\bar{3}m$ ($O_{h}^{5}$)) *γ*-Ag_2_S (*γ*-Ag_1.7_S) silver sulfide at 863 K: *Z* = 4, *a* = *b* = *c* = 0.6270(6) nm.

Atom	Position and Multiplicity	Coordinates of Atoms			Occupancy	*B*_iso_ × 10^−4^ (pm^2^)
		*x*	*y*	*z*		
Ag1	8(*c*)	0.25	0.25	0.25	0.088	0.50
Ag2	32(*j*)	0.33399	0.33399	0.33399	0.15	0.50
Ag3	48(*i*)	0.5	0.30300	0.30300	0.027	0.50
S	4(*a*)	0	0	0	1.00	0.50

**Table 4:** Interatomic (interstitial) spacings *d* for fcc (space group $Fm\bar{3}m$) *γ*-Ag_2_S phase at a 863 K (*a* = 0.62706 nm).

Pair of Atoms (Sites)	CS Number *	Spacing *d* (nm)	Pair of Atoms (Sites)	CS Number *	Spacing *d* (nm)
S—S	1	0.44340	Ag2—Ag2	7	0.25549
4(*a*)—4(*a*)	2	0.62706	32(*j*)—32(*j*)	8	0.25625
Ag1—S	1	0.27152		9	0.25701
8(*c*)—4(*a*)				10	0.29443
Ag2—S	1	0.25549		11	0.29487
32(*j*)—4(*a*)	2	0.25625		12	0.29531
Ag3—S	1	0.22663	Ag2—Ag3	1	0.10775
48(*i*)—4(*a*)			32(*j*)—48(*i*)	2	0.10827
Ag1—Ag1	1	0.31353		3	0.15965
8(*c*)—4(*a*)	2	0.44340		4	0.16039
	3	0.54305		5	0.22662
Ag1—Ag2	1	0.09086		6	0.22696
8(*c*)—32(*j*)	2	0.27117		7	0.22714
	3	0.37317		8	0.25101
Ag1—Ag3	1	0.16366		9	0.25131
8(*c*)—48(*i*)	2	0.32432		10	0.25162
Ag2—Ag2	1	0.14809	Ag3—Ag3	1	0.09400
32(*j*)—32(*j*)	2	0.14853	48(*i*)—48(*i*)	2	0.17470
	3	0.14896		3	0.23617
	4	0.20820		4	0.24706
	5	0.20882		5	0.26870
	6	0.20944		6	0.30259

* CS is coordination sphere.

**Table 5:** Elastic stiffness constants *c_ij_* (GPa), elastic compliance constants *s_ij_* (10^−12^ Pa^−1^), bulk *B*, shear *G*, and the Young’s *E* moduli (GPa), the Poisson’s ratios *μ* and the universal anisotropy criterion *A*^U^ for cubic *β*-Ag_2_S (space group $Im\bar{3}m$) and ZnS (space group $F\bar{4}3m$ ) sulfides at a temperature of 300 K.

Sulfide	*c*_11_	*c*_12_	*c*_44_	*s*_11_	*s*_12_	*s*_44_	*B*_V_ = *B*_R_ = *B*_VRH_	*G*_V_	*G*_R_	*G*_VRH_	*E*	*μ*	*A* ^U^
*β*-Ag_2_S	99.4	7.6	19.0	10.17	−0.72	52.63	38.2	29.8	24.8	27.3	66.1	0.211	1.008
ZnS	93.6	46.6	49.2	15.97	−5.31	20.33	62.3	38.9	34.2	36.1	90.8	0.257	0.687

## Data Availability

The data presented in this study are available on request from the corresponding author.

## Supplementary Materials

The following are available online at https://www.mdpi.com/article/10.3390/nano12101668/s1, Figure S1: (a) The cubic (space group $F\bar{4}3m$) unit cell of ZnS sphalerite: (●) metal sites 4(*a*) occupied by Zn atoms, (●) nonmetal sites 4(*c*) occupied by S atoms; (b) the cubic (space group $Im\bar{3}m$) unit cell of *β*-Ag_2_S argentite: (●) nonmetal sites 2(*a*) occupied by S atoms, (*****) sites 6(*b*) occupied by Ag atoms with probability ~0.0978, and (**X**) sites 48(*j)* filled by Ag atoms with occupation degree ~0.0711. Table S1: Coordinates of all sites for Ag and S atoms in the unit cell of cubic (space group No. 229—$Im\bar{3}m$) silver sulfide with *β*-Ag_2_S argentite-type structure. Figure S2: Arrangement of four Ag atoms in one plane (*z* = 0.5) at one (*b*) type position and three (*j*) type positions of the unit cell of a cubic (space group $Im\bar{3}m$) silver sulfide with argentite *β*-Ag_2_S structure. Figure S3: Two variants of arrangement of four Ag atoms in one plane (*z* = 0.8306 or 0.1694) at four (*j*) type positions of the unit cell of a cubic (space group $Im\bar{3}m$) silver sulfide with argentite *β*-Ag_2_S structure. Figure S4: Arrangement of four Ag atoms in one plane (*z* = 0.3306) at (*j*) type positions of the unit cell of a cubic (space group $Im\bar{3}m$) silver sulfide with argentite *β*-Ag_2_S structure. Figure S5: Arrangement of four Ag atoms in one plane (*z* = 0.6694) at (*j*) type positions of the unit cell of a cubic (space group $Im\bar{3}m$) silver sulfide with argentite *β*-Ag_2_S structure. Figure S6: Arrangement of four Ag atoms in one plane (*z* = 0.4123) at (*j*) type positions of the unit cell of a cubic (space group $Im\bar{3}m$) silver sulfide with argentite *β*-Ag_2_S structure. Figure S7: The EDX spectrum of (Ag_2_S)_0.1_(ZnS) heteronanostructure. The contents of Zn, Ag and S f are equal to 43.5 ± 0.2, 8.7 ± 0.1 and 47.8 ± 0.2 at.% (or 53.4 ± 0.3, 17.6 ± 0.2 and 28.7 ± 0.2 wt. %), respectively. Figure S8: HRTEM image of (Ag_2_S)_0.025_(ZnS) heteronanostructure. Selected areas (*1*)–(*4*) correspond to cubic ZnS sphalerite, and selected area (*5*) corresponds to cubic silver sulfide with *β*-Ag_2_S argentite structure. Figure S9: The EDX spectrum of (Ag_2_S)_0.025_(ZnS) heteronanostructure. The contents of Zn, Ag and S are equal to 48.2 ± 0.3, 2.4 ± 0.1 and 49.4 ± 0.3 at.% (or 63.1 ± 0.3, 5.2 ± 0.1 and 31.7 ± 0.2 wt. %), respectively. Figure S10: The elastic characteristics of monocrystalline ZnS and *β*-Ag_2_S particles as a functions of the crystallographic direction at 300 K: (a) the Young modulus *E*_0*kl*_, (b) the shear modulus *G*_0*kl*_, (c) the bulk modulus *B*_0*kl*_, and (d) the Poisson’s ratio *μ*_0*kl*_ in (100) plane of ZnS sphalerite; (e) the Young modulus *E*_0*kl*_, (f) shear modulus *G*_0*kl*_, (g) bulk modulus *B*_0*kl*_, and (h) the Poisson’s ratio *μ*_0*kl*_ in (100) plane of *β*-Ag_2_S argentite. Distributions of *E_hkl_*, *G_hkl_*, *B_hkl_*, and *μ_hkl_* in (010) and (001) planes have the same shape. Section S2: The bulk *B* and shear *G* moduli as functions of the elastic stiffness constants *c_ij_* and the elastic compliance constants *s_ij_*.
