# Supplementary material for: Polymorphic Phase Transformations in Nanocrystalline Ag2S Silver Sulfide in a Wide Temperature Interval and Influence of Nanostructured Ag2S on the Interface Formation in Ag2S/ZnS Heteronanostructure
Source: Nanomaterials (Basel). 2022 May 13;12(10):1668. doi: 10.3390/nano12101668 (PMC9146971; doi:10.3390/nano12101668)
Supplement: Supplementary file 1 [file nanomaterials-12-01668-s001.zip › nanomaterials-1681802-supplementary.pdf]

Supplementary Material

Polymorphic Phase Transformations in Nanocrystalline Ag_2S Silver Sulfide in a Wide Temperature Interval and Influence of Nanostructured Ag_2S on the Interface Formation in Ag_2S/ZnS Heteronanostructure

Albina A. Valeeva [1,2], Stanislav I. Sadovnikov [1] and Aleksandr I. Gusev [1,*]

[1] Institute of Solid State Chemistry, Ural Branch of the Russian Academy of Sciences, 620990 Ekaterinburg, Russia; anibla_v@mail.ru (A.A.V.); sadovnikov@ihim.uran.ru (S.I.S.)
[2] Ural Federal University named after the first President of Russia B. N. Yeltsin, 620002 Ekaterinburg, Russia
* Correspondence: gusev@ihim.uran.ru

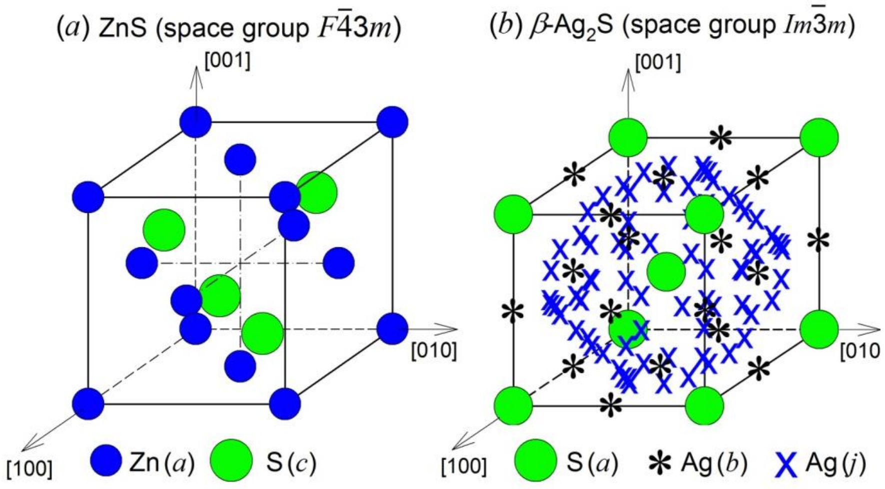

Figure S1. (**a**) The cubic (space group $F\bar{4}3m$) unit cell of ZnS sphalerite: (•) metal sites 4(*a*) occupied by Zn atoms, (•) nonmetal sites 4(*c*) occupied by S atoms; (**b**) the cubic (space group $Im\bar{3}m$) unit cell of β-Ag_2S argentite: (•) nonmetal sites 2(*a*) occupied by S atoms, (*) sites 6(*b*) occupied by Ag atoms with probability ~0.0978, and (**X**) sites 48(*j*) filled by Ag atoms with occupation degree ~0.0711. Figure 1(*b*) is reproduced from Ref. [40] with permission from the PCCP Owner Societies.

Table S1.

Coordinates of all sites for Ag and S atoms in the unit cell of cubic (space group No. 229 - $Im\bar{3}m$ ($I\,4m\,\bar{3}2/m$) (O_h^9)) silver sulfide with β-Ag_2S argentite-type structure (*see* Fig. 1): $Z = 2$, $a = b = c = 0.4874(1)$ nm

Designation of atom and type of position	Derived positions as a sum of initial position and vector	Atomic coordinates in the unit cell			Occupancy
		x	*y*	*z*	
Ag1 (*j*)		0.3306(5)	0.4122(7)	0	0.0711(0)
Ag2 (*j*)		0.6694(5)	0.4122(7)	0	0.0711(0)
Ag3 (*j*)		0.3306(5)	0.5878(3)	0	0.0711(0)
Ag4 (*j*)		0.6694(5)	0.5878(3)	0	0.0711(0)
Ag5 (*j*)		0.4122(7)	0.3306(5)	0	0.0711(0)
Ag6 (*j*)		0.4122(7)	0.6694(5)	0	0.0711(0)
Ag7 (*j*)		0.5878(3)	0.3306(5)	0	0.0711(0)
Ag8 (*j*)		0.5878(3)	0.6694(5)	0	0.0711(0)
Ag9 (*j*)	24(*j*) +(1 0 0)	1	0.3306(5)	0.4122(7)	0.0711(0)
Ag10 (*j*)	23(*j*) +(1 0 0)	1	0.4122(7)	0.3306(5)	0.0711(0)
Ag11 (*j*)	26(*j*) +(0 1 0)	0.5878(3)	1	0.3306(5)	0.0711(0)
Ag12 (*j*)	20(*j*) +(0 1 0)	0.3306(5)	1	0.4122(7)	0.0711(0)
Ag13 (*j*)	3(*j*) +(1/2 1/2 1/2)	0.8306(5)	0.0878(3)	0.5	0.0711(0)
Ag14 (*j*)	22(*j*) +(1/2 1/2 1/2)	0.5	0.9122(7)	0.1694(5)	0.0711(0)
Ag15 (*j*)	2(*j*) +(1/2 1/2 1/2)	0.1694(5)	0.9122(7)	0.5	0.0711(0)
Ag16 (*j*)		0	0.3306(5)	0.5878(3)	0.0711(0)
Ag17 (*j*)		0	0.6694(5)	0.5878(3)	0.0711(0)
Ag18 (*j*)		0.4122(7)	0	0.3306(5)	0.0711(0)
Ag19 (*j*)		0.4122(7)	0	0.6694(5)	0.0711(0)

Ag20 (*j*)		0.3306(5)	0	0.4122(7)	0.0711(0)
Ag21 (*j*)		0.6694(5)	0	0.4122(7)	0.0711(0)
Ag22 (*j*)		0	0.4122(7)	0.6694(5)	0.0711(0)
Ag23 (*j*)		0	0.4122(7)	0.3306(5)	0.0711(0)
Ag24 (*j*)		0	0.3306(5)	0.4122(7)	0.0711(0)
Ag25 (*j*)		0	0.6694(5)	0.4122(7)	0.0711(0)
Ag26 (*j*)		0.5878(3)	0	0.3306(5)	0.0711(0)
Ag27 (*j*)		0.5878(3)	0	0.6694(5)	0.0711(0)
Ag28 (*j*)		0.3306(5)	0	0.5878(3)	0.0711(0)
Ag29 (*j*)		0.6694(5)	0	0.5878(3)	0.0711(0)
Ag30 (*j*)		0	0.5878(3)	0.6694(5)	0.0711(0)
Ag31 (*j*)		0	0.5878(3)	0.3306(5)	0.0711(0)
Ag32 (*j*)	25(*j*) +(1 0 0)	1	0.6694(5)	0.4122(7)	0.0711(0)
Ag33 (*j*)	16(*j*) +(1 0 0)	1	0.3306(5)	0.5878(3)	0.0711(0)
Ag34 (*j*)	17(*j*) +(1 0 0)	1	0.6694(5)	0.5878(3)	0.0711(0)
Ag35 (*j*)	22(*j*) +(1 0 0)	1	0.4122(7)	0.6694(5)	0.0711(0)
Ag36 (*j*)	30(*j*) +(1 0 0)	1	0.5878(3)	0.6694(5)	0.0711(0)
Ag37 (*j*)	31(*j*) +(1 0 0)	1	0.5878(3)	0.3306(5)	0.0711(0)
Ag38 (*j*)	18(*j*) +(0 1 0)	0.4122(7)	1	0.3306(5)	0.0711(0)
Ag39 (*j*)	19(*j*) +(0 1 0)	0.4122(7)	1	0.6694(5)	0.0711(0)
Ag40 (*j*)	27(*j*) +(0 1 0)	0.5878(3)	1	0.6694(5)	0.0711(0)
Ag41 (*j*)	28(*j*) +(0 1 0)	0.3306(5)	1	0.5878(3)	0.0711(0)
Ag42 (*j*)	29(*j*) +(0 1 0)	0.6694(5)	1	0.5878(3)	0.0711(0)
Ag43 (*j*)	21(*j*) +(0 1 0)	0.6694(5)	1	0.4122(7)	0.0711(0)
Ag44 (*j*)	23(*j*) +(1/2 1/2 1/2)	0.5	0.9122(7)	0.8306(5)	0.0711(0)
Ag45 (*j*)	30(*j*) +(1/2 1/2 1/2)	0.5	0.0878(3)	0.1694(5)	0.0711(0)
Ag46 (*j*)	31(*j*) +(1/2 1/2 1/2)	0.5	0.0878(3)	0.8306(5)	0.0711(0)
Ag47 (*j*)	1(*j*) +(1/2 1/2 1/2)	0.8306(5)	0.9122(7)	0.5	0.0711(0)
Ag48 (*j*)	4(*j*) +(1/2 1/2 1/2)	0.1694(5)	0.0878(3)	0.5	0.0711(0)
Ag49 (*j*)	1(*j*) +(0 0 1)	0.3306(5)	0.4122(7)	1	0.0711(0)
Ag50 (*j*)	2(*j*) +(0 0 1)	0.6694(5)	0.4122(7)	1	0.0711(0)
Ag51 (*j*)	3(*j*) +(0 0 1)	0.3306(5)	0.5878(3)	1	0.0711(0)
Ag52 (*j*)	4(*j*) +(0 0 1)	0.6694(5)	0.5878(3)	1	0.0711(0)
Ag53 (*j*)	5(*j*) +(0 0 1)	0.4122(7)	0.3306(5)	1	0.0711(0)
Ag54 (*j*)	6(*j*) +(0 0 1)	0.4122(7)	0.6694(5)	1	0.0711(0)
Ag55 (*j*)	7(*j*) +(0 0 1)	0.5878(3)	0.3306(5)	1	0.0711(0)
Ag56 (*j*)	8(*j*) +(0 0 1)	0.5878(3)	0.6694(5)	1	0.0711(0)
Ag57 (*j*)	24(*j*) +(1/2 1/2 1/2)	0.5	0.8306(5)	0.9122(7)	0.0711(0)
Ag58 (*j*)	25(*j*) +(1/2 1/2 1/2)	0.5	0.1694(5)	0.9122(7)	0.0711(0)
Ag59 (*j*)	16(*j*) +(1/2 1/2 1/2)	0.5	0.8306(5)	0.0878(3)	0.0711(0)
Ag60 (*j*)	17(*j*) +(1/2 1/2 1/2)	0.5	0.1694(5)	0.0878(3)	0.0711(0)
Ag61 (*j*)	18(*j*) +(1/2 1/2 1/2)	0.9122(7)	0.5	0.8306(5)	0.0711(0)
Ag62 (*j*)	19(*j*) +(1/2 1/2 1/2)	0.9122(7)	0.5	0.1694(5)	0.0711(0)
Ag63 (*j*)	26(*j*) +(1/2 1/2 1/2)	0.0878(3)	0.5	0.8306(5)	0.0711(0)
Ag64 (*j*)	27(*j*) +(1/2 1/2 1/2)	0.0878(3)	0.5	0.1694(5)	0.0711(0)
Ag65 (*j*)	28(*j*) +(1/2 1/2 1/2)	0.8306(5)	0.5	0.0878(3)	0.0711(0)
Ag66 (*j*)	29(*j*) +(1/2 1/2 1/2)	0.1694(5)	0.5	0.0878(3)	0.0711(0)
Ag67 (*j*)	12(*j*) +(1/2 1/2 1/2)	0.8306(5)	0.5	0.9122(7)	0.0711(0)
Ag68 (*j*)	21(*j*) +(1/2 1/2 1/2)	0.1694(5)	0.5	0.9122(7)	0.0711(0)
Ag69 (*j*)	5(*j*) +(1/2 1/2 1/2)	0.9122(7)	0.8306(5)	0.5	0.0711(0)
Ag70 (*j*)	6(*j*) +(1/2 1/2 1/2)	0.9122(7)	0.1694(5)	0.5	0.0711(0)
Ag71 (*j*)	7(*j*) +(1/2 1/2 1/2)	0.0878(3)	0.8306(5)	0.5	0.0711(0)

Ag72 (*j*)	8(*j*) +(1/2 1/2 1/2)	0.0878(3)	0.1694(5)	0.5	0.0711(0)
Ag73 (*b*)		0.5	0.5	0	0.0978(7)
Ag74 (*b*)	76(*b*) +(1/2 1/2 1/2)	0.5	0	0	0.0978(7)
Ag75 (*b*)	77(*b*) +(1/2 1/2 1/2)	0	0.5	0	0.0978(7)
Ag76 (*b*)		0	0.5	0.5	0.0978(7)
Ag77 (*b*)		0.5	0	0.5	0.0978(7)
Ag78 (*b*)	73(*b*) +(1/2 1/2 1/2)	0	0	0.5	0.0978(7)
Ag79 (*b*)	76(*b*) +(1 0 0)	1	0.5	0.5	0.0978(7)
Ag80 (*b*)	75(*b*) +(1 0 0)	1	0.5	0	0.0978(7)
Ag81 (*b*)	75(*b*) +(0 0 1)	0	0.5	1	0.0978(7)
Ag82 (*b*)	77(*b*) +(0 1 0)	0.5	1	0.5	0.0978(7)
Ag83 (*b*)	78(*b*) +(1 0 0)	1	0	0.5	0.0978(7)
Ag84 (*b*)	78(*b*) +(0 1 0)	0	1	0.5	0.0978(7)
Ag85 (*b*)	78(*b*) +(1 1 0)	1	1	0.5	0.0978(7)
Ag86 (*b*)	73(*b*) +(0 0 1)	0.5	0.5	1	0.0978(7)
Ag87 (*b*)	74(*b*) +(0 1 1)	0.5	1	1	0.0978(7)
Ag88 (*b*)	74(*b*) +(0 1 0)	0.5	1	0	0.0978(7)
Ag89 (*b*)	74(*b*) +(0 0 1)	0.5	0	1	0.0978(7)
Ag90 (*b*)	75(*b*) +(1 0 1)	1	0.5	1	0.0978(7)
S1 (*a*)		0	0	0	1.00(0)
S2 (*a*)	1(*a*)+ (1/2 1/2 1/2)	0.5	0.5	0.5	1.00(0)
S3 (*a*)	1(*a*) +(1 0 0)	1	0	0	1.00(0)
S4 (*a*)	1(*a*) +(0 1 0)	0	1	0	1.00(0)
S5 (*a*)	1(*a*) +(0 0 1)	0	0	1	1.00(0)
S6 (*a*)	1(*a*) +(1 0 1)	1	0	1	1.00(0)
S7 (*a*)	1(*a*) +(0 1 1)	0	1	1	1.00(0)
S8 (*a*)	1(*a*) +(1 1 0)	1	1	0	1.00(0)
S9 (*a*)	1(*a*) +(1 1 1)	1	1	1	1.00(0)

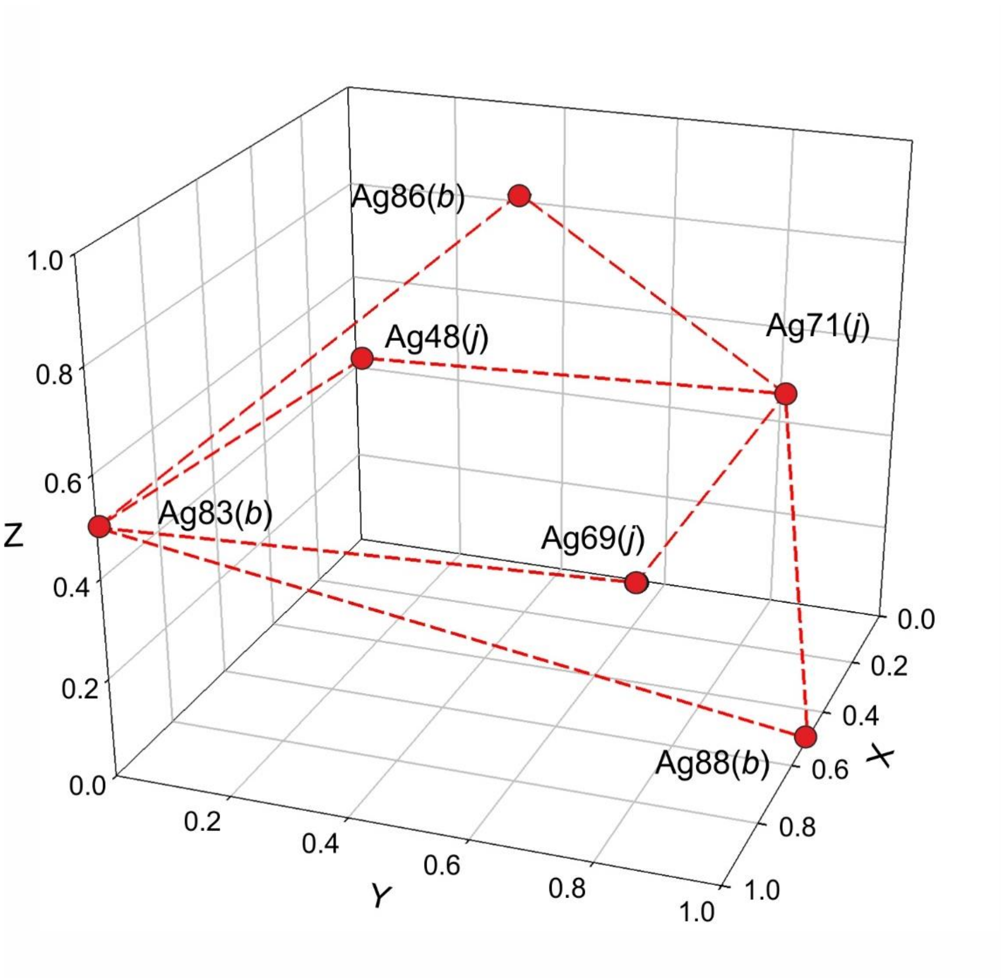

Figure S2. Arrangement of four Ag atoms in one plane (z = 0.5) at one (b) type position and three (j) type positions of the unit cell of a cubic (space group $Im\bar{3}m$) silver sulfide with argentite β-Ag_2S structure.

Atom	x	y	z		Pair of Ag atoms	Interatomic distance d (Å)
Ag83 (b)	1.0	0	0.5		Ag83(b) – Ag48(j)	4.0604
Ag48 (j)	0.1694	0.0878	0.5		Ag48(j) – Ag71(j)	3.6328
Ag71 (j)	0.0878	0.8306	0.5		Ag71(j) – Ag69(j)	4.0077
Ag69 (j)	0.9122	0.8306	0.5		Ag69(j) – Ag83(b)	4.0604
Ag83 (b)	1.0	0	0.5		Ag83(b) – Ag86(b)	4.2101
Ag86 (b)	0.5	0.5	1		Ag86(b) – Ag71(j)	3.5365
Ag71 (j)	0.0878	0.8306	0.5		Ag71(j) – Ag88(b)	3.2561
Ag88 (b)	0.5	1.0	0		Ag88(b) – Ag83(b)	5.9540

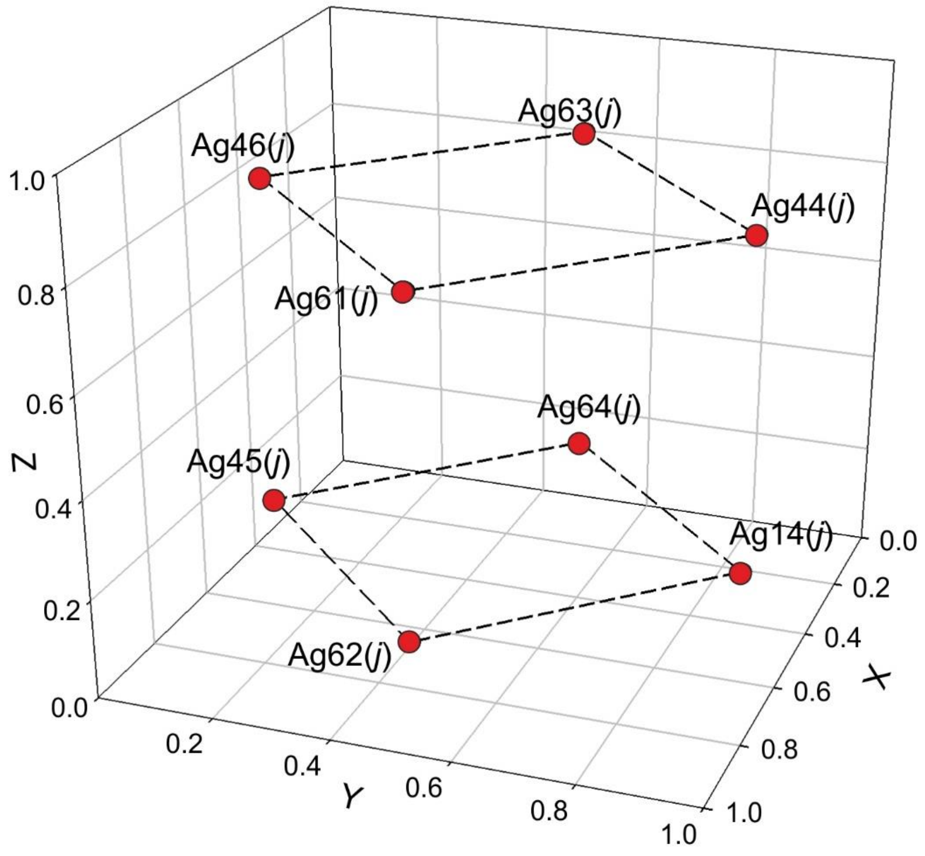

Figure S3. Two variants of arrangement of four Ag atoms in one plane (z = 0.8306 or 0.1694) at four (j) type positions of the unit cell of a cubic (space group $Im\bar{3}m$) silver sulfide with argentite β-Ag_2S structure.

Atom	*x*	*y*	*Z*		Pair of Ag atoms	Interatomic distance *d* (Å)
Ag61 (*j*)	0.9122	0.5	0.8306		Ag61(*j*) – Ag44(*j*)	2.8344
Ag44 (*j*)	0.5	0.9122	0.8306		Ag44(*j*) – Ag63(*j*)	2.8344
Ag63 (*j*)	0.0877	0.5	0.8306		Ag63(*j*) – Ag46(*j*)	2.8344
Ag46 (*j*)	0.5	0.0877	0.8306		Ag46(*j*) – Ag61(*j*)	2.8344

Atom	*x*	*y*	*z*		Pair of Ag atoms	Interatomic distance *d* (Å)
Ag62 (*j*)	0.9122	0.5	0.1694		Ag62(*j*) - Ag14(*j*)	2.8344
Ag14 (*j*)	0.5	0.9122	0.1694		Ag14(*j*) - Ag64(*j*)	2.8344
Ag64 (*j*)	0.0878	0.5	0.1694		Ag64(*j*) - Ag45(*j*)	2.8344
Ag45 (*j*)	0.5	0.0878	0.1694		Ag45(*j*) - Ag62(*j*)	2.8344

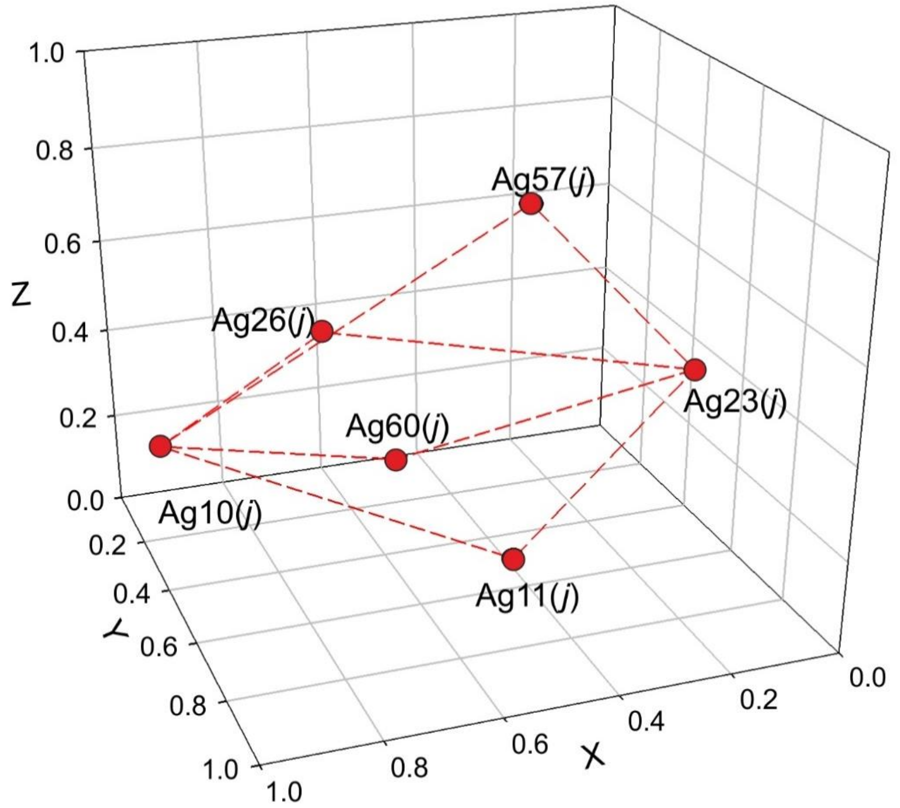

Figure S4. Arrangement of four Ag atoms in one plane (*z* = 0.3306) at (*j*) type positions of unit cell of a cubic (space group $Im\bar{3}m$) silver sulfide with argentite *β*-Ag_2S structure.

Atom	*x*	*y*	*z*		Pair of Ag atoms	Interatomic distance *d* (Å)
Ag23 (*j*)	0	0.4123	0.3306		Ag23(*j*) - Ag26(*j*)	3.4900
Ag26 (*j*)	0.5878	0	0.3306		Ag26(*j*) - Ag10(*j*)	2.8344
Ag10 (*j*)	1.0	0.4123	0.3306		Ag10(*j*) – Ag11(*j*)	4.8614
Ag11 (*j*)	0.5878	1.0	0.3306		Ag11(*j*) - Ag23(*j*)	3.4900
Ag23 (*j*)	0	0.4123	0.3306		Ag23(*j*) – Ag57(*j*)	4.2473
Ag57 (*j*)	0.5	0.8306	0.9122		Ag57(*j*) - Ag10(*j*)	4.2473
Ag10 (*j*)	1.0	0.4123	0.3306		Ag10(*j*) – Ag60(*j*)	2.9491
Ag60 (*j*)	0.5	0.1694	0.0878		Ag60(*j*) - Ag23(*j*)	2.9491

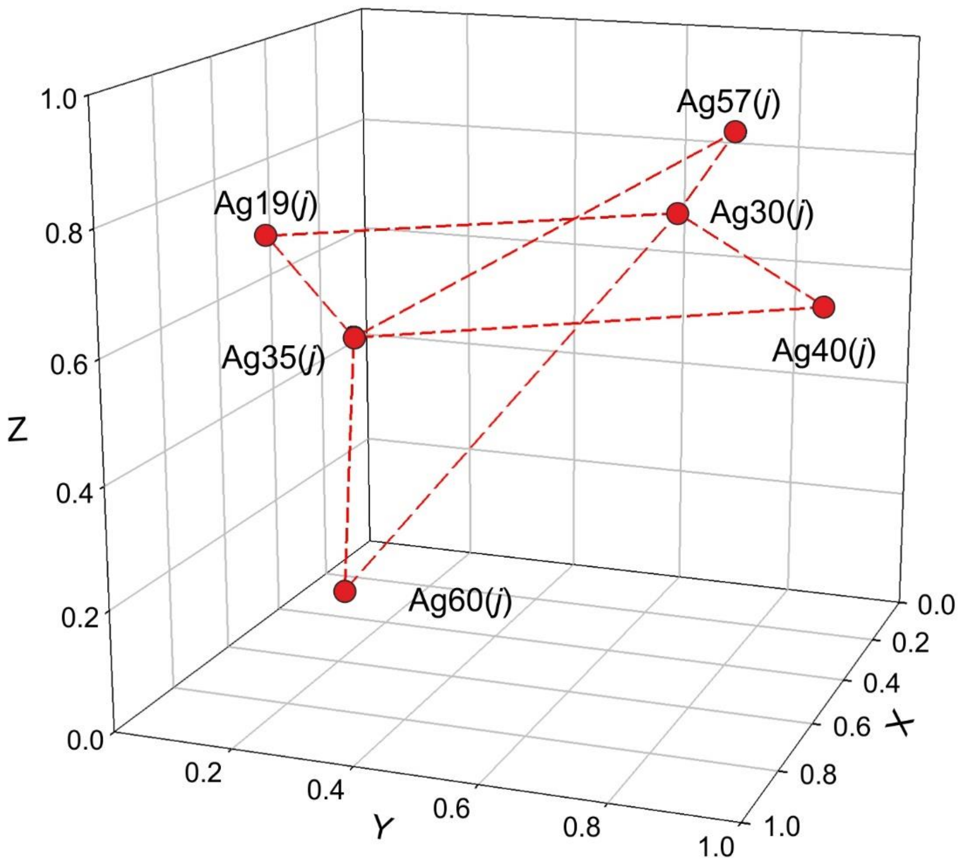

Figure S5. Arrangement of four Ag atoms in one plane (z = 0.6694) at (j) type positions of unit cell of a cubic (space group $Im\bar{3}m$) silver sulfide with argentite β-Ag_2S structure.

Atom	x	y	z		Pair of Ag atoms	Interatomic distance d (Å)
Ag35 (j)	1.0	0.4123	0.6694		Ag35(j) – Ag19(j)	3.4900
Ag19 (j)	0.4123	0	0.6694		Ag19(j) – Ag30(j)	3.4900
Ag30 (j)	0	0.5877	0.6694		Ag30(j) - Ag40(j)	3.4900
Ag40 (j)	0.5877	1.0	0.6694		Ag40(j) – Ag35(j)	3.4900
Ag35 (j)	1.0	0.4123	0.6694		Ag35(j) – Ag57(j)	3.3820
Ag57 (j)	0.5	0.8306	0.9122		Ag57(j) – Ag30(j)	2.9491
Ag30 (j)	0	0.5877	0.6694		Ag30(j) – Ag60(j)	4.2471
Ag60 (j)	0.5	0.1694	0.0878		Ag60(j) – Ag35(j)	3.9111

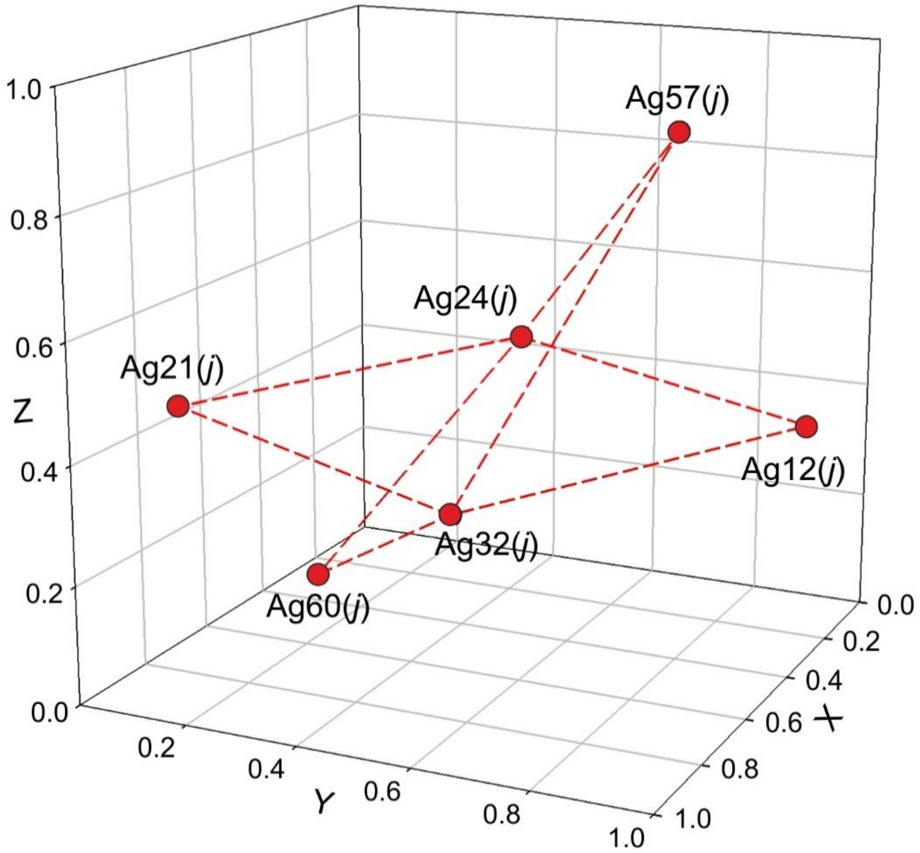

Figure S6. Arrangement of four Ag atoms in one plane (z = 0.4123) at (j) type positions of unit cell of a cubic (space group $Im\bar{3}m$) silver sulfide with argentite β-Ag_2S structure.

Atom	x	y	Z		Pair of Ag atoms	Interatomic distance d (Å)
Ag32 (j)	1.0	0.6694	0.4123		Ag32(j) – Ag21(j)	3.6295
Ag21 (j)	0.6694	0	0.4123		Ag21(j) – Ag24(j)	3.6295
Ag24 (j)	0	0.3306	0.4123		Ag24(j) – Ag12(j)	3.6295
Ag12 (j)	0.3306	1.0	0.4123		Ag12(j) – Ag32(j)	3.6295
Ag32 (j)	1.0	0.6694	0.4123		Ag32(j) – Ag57(j)	3.5257
Ag57 (j)	0.5	0.8306	0.9122		Ag57(j) – Ag24(j)	4.2101
Ag24 (j)	0	0.3306	0.4123		Ag24(j) – Ag60(j)	3.0021
Ag60 (j)	0.5	0.1694	0.0878		Ag60(j) – Ag32(j)	3.7824

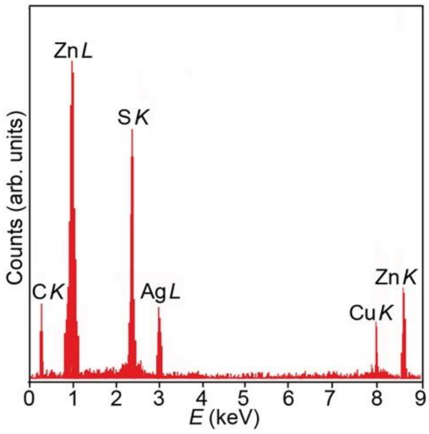

Figure S7. The EDX spectrum of $(Ag_2S)_{0.1}(ZnS)$ heteronanostructure. The contents of Zn, Ag and S f are equal to 43.5±0.2, 8.7±0.1 and 47.8±0.2 at.% (or 53.4±0.3, 17.6±0.2 and 28.7±0.2 wt.%), respectively.

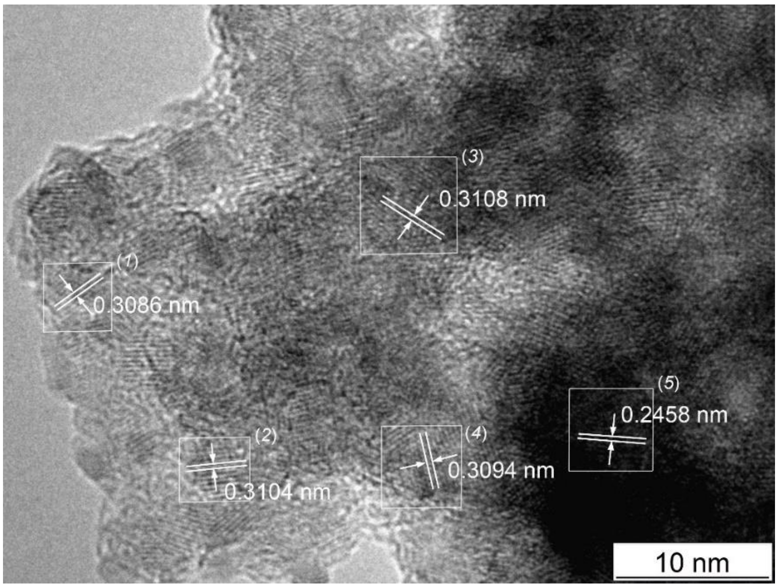

Figure S8. HRTEM image of $(Ag_2S)_{0.025}(ZnS)$ heteronanostructure. Selected areas (*1*)-(*4*) correspond to cubic ZnS sphalerite, and selected area (*5*) corresponds to cubic silver sulfide with β-Ag_2S argentite structure.

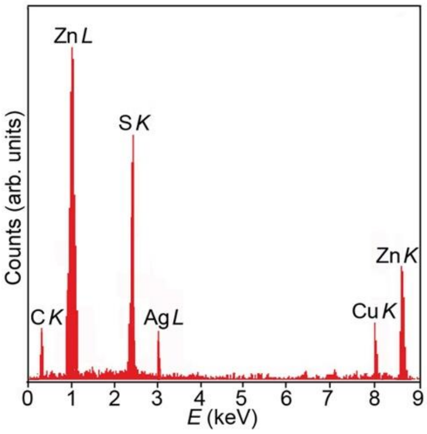

Figure S9. The EDX spectrum of $(Ag_2S)_{0.025}(ZnS)$ heteronanostructure. The contents of Zn, Ag and S are equal to 48.2±0.3, 2.4±0.1 and 49.4±0.3 at.% (or 63.1±0.3, 5.2±0.1 and 31.7±0.2 wt.%), respectively.

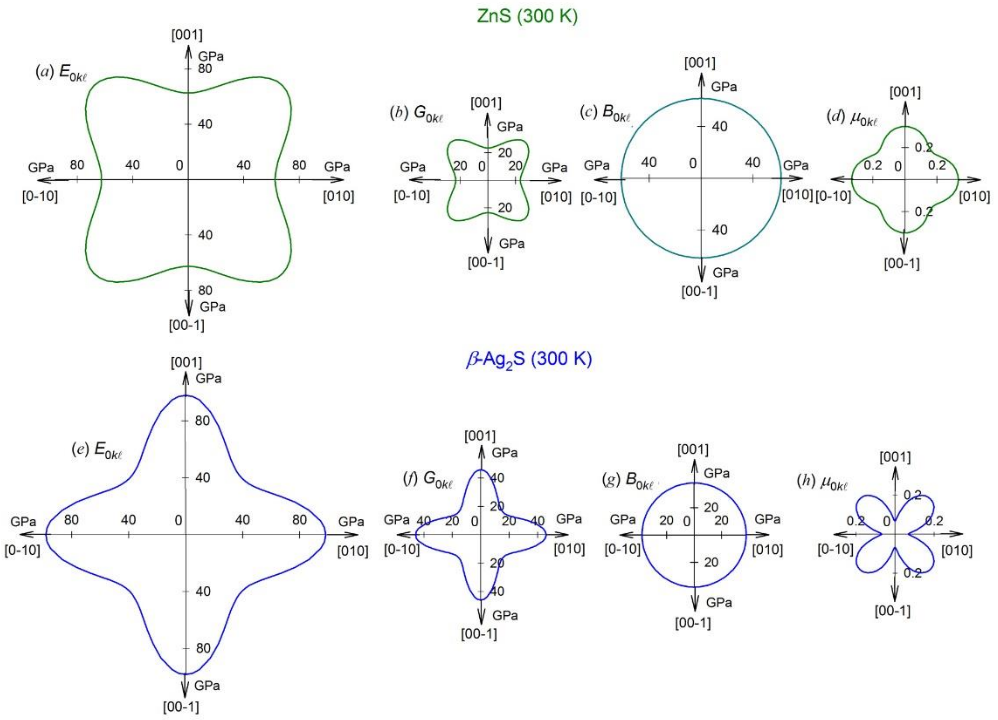

Figure S10. The elastic characteristics of monocrystalline ZnS and β-Ag_2S particles as a functions of the crystallographic direction at 300 K: (**a**) the Young modulus E_{0kl}, (**b**) the shear modulus G_{0kl}, (**c**) the bulk modulus B_{0kl}, and (**d**) the Poisson's ratio μ_{0kl} in (100) plane of ZnS sphalerite; (**e**) the Young modulus E_{0kl}, (**f**) shear modulus G_{0kl}, (**g**) bulk modulus B_{0kl}, and (**h**) the Poisson's ratio μ_{0kl} in (100) plane of β-Ag_2S argentite. Distributions of E_{hkl}, G_{hkl}, B_{hkl}, and μ_{hkl} in (010) and (001) planes have the same shape.

Section S2. The bulk B and shear G moduli as functions of the elastic stiffness constants c_{ij} and the elastic compliance constants s_{ij}

For any crystal system, the bulk and shear moduli are given by the Voigt [89] and Reuss [90] equations where B_V and B_R are the upper and lower limits of bulk modulus B, and G_V and G_R are the upper and lower limits of shear modulus G, respectively:

$$B_V = [c_{11}+c_{22}+c_{33} + 2(c_{12} + c_{13} + c_{23})]/9, \quad (1a)$$

$$B_R = 1/[s_{11} + s_{22} + s_{33} + 2(s_{12} + s_{13} + s_{23})], \quad (1b)$$

$$G_V = [c_{11}+c_{22}+c_{33}+ 3(c_{44}+c_{55}+c_{66}) – (c_{12}+c_{13}+c_{23})]/15 \quad (1c)$$

$$G_R = 15/[4(s_{11}+s_{22}+s_{33}) –4(s_{12}+s_{13}+s_{23}) + 3(s_{44}+ s_{55}+ s_{66})]. \quad (1d)$$

Thus, the models of Voigt [89] and Reuss [90] lead to the theoretical maximum and minimum values of the isotropic elastic modulus, respectively.

Relations (1*a*)-(1*d*) for cubic crystals are simplified and have the form [88]:

$$B_V = (c_{11} + 2c_{12})/3, \quad (2a)$$

$$B_R = 1/[3(s_{11} + 2s_{12})], \quad (2b)$$

$$G_V = (c_{11} + 3c_{44} - c_{12})/5, \quad (8c)$$

$$G_R = 5/[4(s_{11} – s_{12}) + 3s_{44}] \equiv 5c_{44}(c_{11} – c_{12})/[4c_{44} + 3(c_{11} – c_{12})]. \quad (8d)$$

As $c_{11} + 2c_{12} = 1/(s_{11} + 2s_{12})$, it is easily seen that the bulk modulus of cubic crystals is $B_V = B_R$.
